# Exploring the Bimodal Solar System via Sample Return from the Main Asteroid Belt: The Case for Revisiting Ceres

**DOI:** 10.1007/s11214-020-00671-0

**Published:** 2020-05-18

**Authors:** Thomas H. Burbine, Richard C. Greenwood

**Affiliations:** 1 Department of Astronomy, Mount Holyoke College, South Hadley, MA 01075 USA; 2 Planetary and Space Sciences, School of Physical Sciences, The Open University, Walton Hall, Milton Keynes, MK7 6AA UK

**Keywords:** Asteroid, Sample return, Main belt, Ceres, Meteorites, Spacecraft

## Abstract

Sample return from a main-belt asteroid has not yet been attempted, but appears technologically feasible. While the cost implications are significant, the scientific case for such a mission appears overwhelming. As suggested by the “Grand Tack” model, the structure of the main belt was likely forged during the earliest stages of Solar System evolution in response to migration of the giant planets. Returning samples from the main belt has the potential to test such planet migration models and the related geochemical and isotopic concept of a bimodal Solar System.

Isotopic studies demonstrate distinct compositional differences between samples believed to be derived from the outer Solar System (CC or carbonaceous chondrite group) and those that are thought to be derived from the inner Solar System (NC or non-carbonaceous group). These two groups are separated on relevant isotopic variation diagrams by a clear compositional gap. The interface between these two regions appears to be broadly coincident with the present location of the asteroid belt, which contains material derived from both groups.

The Hayabusa mission to near-Earth asteroid (NEA) (25143) Itokawa has shown what can be learned from a sample-return mission to an asteroid, even with a very small amount of sample. One scenario for main-belt sample return involves a spacecraft launching a projectile that strikes an object and flying through the debris cloud, which would potentially allow multiple bodies to be sampled if a number of projectiles are used on different asteroids. Another scenario is the more traditional method of landing on an asteroid to obtain the sample.

A significant range of main-belt asteroids are available as targets for a sample-return mission and such a mission would represent a first step in mineralogically and isotopically mapping the asteroid belt. We argue that a sample-return mission to the asteroid belt does not necessarily have to return material from both the NC and CC groups to viably test the bimodal Solar System paradigm, as material from the NC group is already abundantly available for study. Instead, there is overwhelming evidence that we have a very incomplete suite of CC-related samples.

Based on our analysis, we advocate a dedicated sample-return mission to the dwarf planet (1) Ceres as the best means of further exploring inherent Solar System variation. Ceres is an ice-rich world that may be a displaced trans-Neptunian object. We almost certainly do not have any meteorites that closely resemble material that would be brought back from Ceres. The rich heritage of data acquired by the Dawn mission makes a sample-return mission from Ceres logistically feasible at a realistic cost. No other potential main-belt target is capable of providing as much insight into the early Solar System as Ceres. Such a mission should be given the highest priority by the international scientific community.

## Introduction

### Why Is Sample Return Important?

Thanks to the regular delivery of meteorite samples to Earth, we already have an extensive and diverse range of relatively pristine extraterrestrial materials in our worldwide collections. These falls are augmented by an even more extensive inventory of meteorite finds, mainly recovered from hot (e.g., Sahara) and cold (e.g., Antarctica) desert regions. The result is that there are currently more than 60,000 individual meteorite samples (Meteoritical Bulletin Database 2020) available on Earth for analysis. In addition to meteorites, we also have significant collections of Interplanetary Dust Particles (IDPs) and micrometeorites collected in a diverse range of terrestrial environments, including the stratosphere, deep ocean basins, and polar regions (Brownlee 1985; Bradley 2014; Noguchi et al. 2015).

With such a diverse assemblage of, essentially low-cost, extraterrestrial materials available for detailed characterization studies, it is a valid question to ask why significantly more expensive samples need to be collected by robotic missions.

The problem is that apart from relatively rare fireball trajectory information (Devillepoix et al. 2018), and one-off events, such as the tracking of asteroid 2008 TC_3_ prior to atmospheric entry (Shaddad et al. 2010), most Earth-recovered extraterrestrial materials provide little detailed information about which bodies such samples actually originated from. In contrast, collecting material directly from a well characterized source body, as was the case of asteroid (25143) Itokawa (Nakamura et al. 2011), provides a geologic context that is unavailable for Earth-recovered samples. A very important aspect of these sample-return missions is that they link together laboratory (e.g., isotopic analyses, mineralogy) and remote sensing (e.g., images, reflectance spectra) data.

Another problem with the extraterrestrial samples recovered on Earth is that they are likely to be a very unrepresentative and biased sampling of Solar System bodies (Campbell-Brown 2019). Very friable carbonaceous materials are likely under-represented in the meteorite record as they tend not to be able to survive atmospheric entry intact (e.g., Sears 1998). Poorly consolidated meteorites, such as the ungrouped carbonaceous chondrite Tagish Lake, are rare exceptions (Brown et al. 2000).

Sample return is not a new concept and in fact has already made a very significant contribution to our understanding of Solar System evolution. It is not an exaggeration to say that some of the greatest engineering feats of the late 20th and early 21st century have been sample-return missions from planetary bodies. Samples have been returned from the Moon (Apollo and Luna missions) (e.g., Lunar Sample Preliminary Examination Team 1969), a comet [Stardust mission to comet 81P/Wild (Wild 2)] (e.g., Zolensky et al. 2006), and a near-Earth asteroid (NEA) [Hayabusa mission to (25143) Itokawa] (e.g., Nakamura et al. 2011). Solar wind particles have also been collected from space by the Genesis mission (e.g., Grimberg et al. 2006). Two NEA sample-return missions are currently being conducted: the Hayabusa2 spacecraft to (162173) Ryugu (e.g., Wada et al. 2018; Watanabe et al. 2019) and the OSIRIS-REx spacecraft to (101955) Bennu (e.g., Bierhaus et al. 2018; Lauretta et al. 2019). Both asteroids are C-complex bodies. C-complex asteroids tend to have low albedos, absorption features (when present) due to hydrated silicates, and have been typically linked with carbonaceous chondrites. A Japanese sample-return mission (MMX: Martian Moons eXploration) to Phobos is currently being planned (Usui et al. 2020). Except for the Apollo missions, where the sampling was undertaken by humans, all sample-return missions so far have been done robotically.

These sample-return missions have helped to “solve” a number of planetary science questions. Lunar samples have given evidence for an impact origin for the Moon (Hartmann and Davis 1975), the presence of an early magma ocean (Wood et al. 1970), and the likely existence of a relatively high water content in the lunar interior (Saal et al. 2008). Samples from comet Wild 2 have revealed the extent to which mixing of high temperature solids took place throughout the disk during the earliest stages of Solar System evolution (Zolensky et al. 2006; Brownlee 2014; Westphal et al. 2017). Analyses of samples collected by Hayabusa show evidence for space weathering (Noguchi et al. 2011) and confirm that at least one S-complex asteroid has an ordinary chondrite composition (Nakamura et al. 2011). S-complex bodies have absorption features that tend to be due to olivine and/or pyroxene and have been typically linked with ordinary chondrites. Analyses of solar wind samples collected by the Genesis mission have allowed the oxygen isotopic composition of the Sun to be estimated (McKeegan et al. 2011).

### Why do We Need Samples from the Asteroid Belt?

While NEAs have been sampled by the Hayabusa mission (Nakamura et al. 2011) and are currently the targets of Hayabusa2 and OSIRIS-REx missions (Watanabe et al. 2019; Lauretta et al. 2019), what has so far never been attempted is a sample-return mission from a main-belt object, which have semi-major axes ($a$) between ∼2.1 and ∼3.3 AU. While the cost implications of such a mission are significant, the scientific case for it is now overwhelming. The reason for this is that potentially the main belt holds the key to a more profound understanding of early Solar System evolution. This is because the structure of the main belt was likely forged during the earliest stages of Solar System history, most probably in response to migration of the giant planets (Walsh et al. 2011, 2012). This migration would have scattered bodies into their present-day locations in the asteroid belt.

Returning samples from a main-belt asteroid has the potential, not only to test current planet migration models (e.g., Walsh et al. 2011, 2012), but also to furnish critical evidence with which to test current geochemical concepts of a bimodal Solar System (e.g., Warren 2011; Kruijer et al. 2017, 2020; Scott et al. 2018; Kleine et al. 2020) by mineralogically and isotopically mapping the asteroid belt. This bimodality is in the form of compositional and isotopic differences between materials that most likely originated in the inner Solar System compared to those that probably formed in the outer Solar System (Warren 2011). As we will discuss, models for the early dynamical evolution of the main belt are inextricably linked to the concept of such a bimodal Solar System (Morbidelli et al. 2015). The central goal of a sample-return mission to the main belt would be to rigorously test models of Solar System formation. The return and subsequent detailed laboratory analyses of samples collected directly from main-belt asteroids represent the most fundamental approach available, both in terms of testing our existing migration models and also obtaining critical data that may lead to the development of radically new ideas about the formation of our Solar System and its early evolution. The latest planetary decadal survey (National Research Council 2011) states that one of the important questions to try to answer in the future is “What were the initial stages, conditions, and processes of Solar System formation and the nature of the interstellar matter that was incorporated?”

The problem with sample return from just NEAs is that these bodies have dynamical lifetimes of only a few million years (Gladman et al. 1997) and are fragments derived from much larger bodies. The probability that a NEA or meteorite with a known orbit originates from a particular source region (e.g., Hungaria region, $\nu _{6}$ secular resonance, 3:1 mean motion resonance) (e.g., Bottke et al. 2002a; Granvik and Brown 2018) can be calculated; however, it is currently not possible to definitively link any NEA or meteorite with any main-belt object. Therefore, sample return from a NEA does not give any definitive information on the exact location in the main belt where the NEA originated.

In this paper, we will look in detail at why sample return from the main belt is a critical next step in the exploration of the Solar System and has the potential to significantly improve our understanding of its early evolution. We will look first at the present-day structure of the asteroid belt and review dynamical models for its evolution. We will then examine recent geochemical concepts that invoke an essentially bimodal composition for Solar System materials. We will discuss the importance of sample return from main-belt objects and possible scenarios for bringing fragments of these bodies back to Earth. We will examine possible destinations and finally present what we believe is the single most strategic target to “solve” the bimodal Solar System paradigm.

## Structure of the Main Belt and Its Link to Gas Giant Migration

**Fig. 1:**
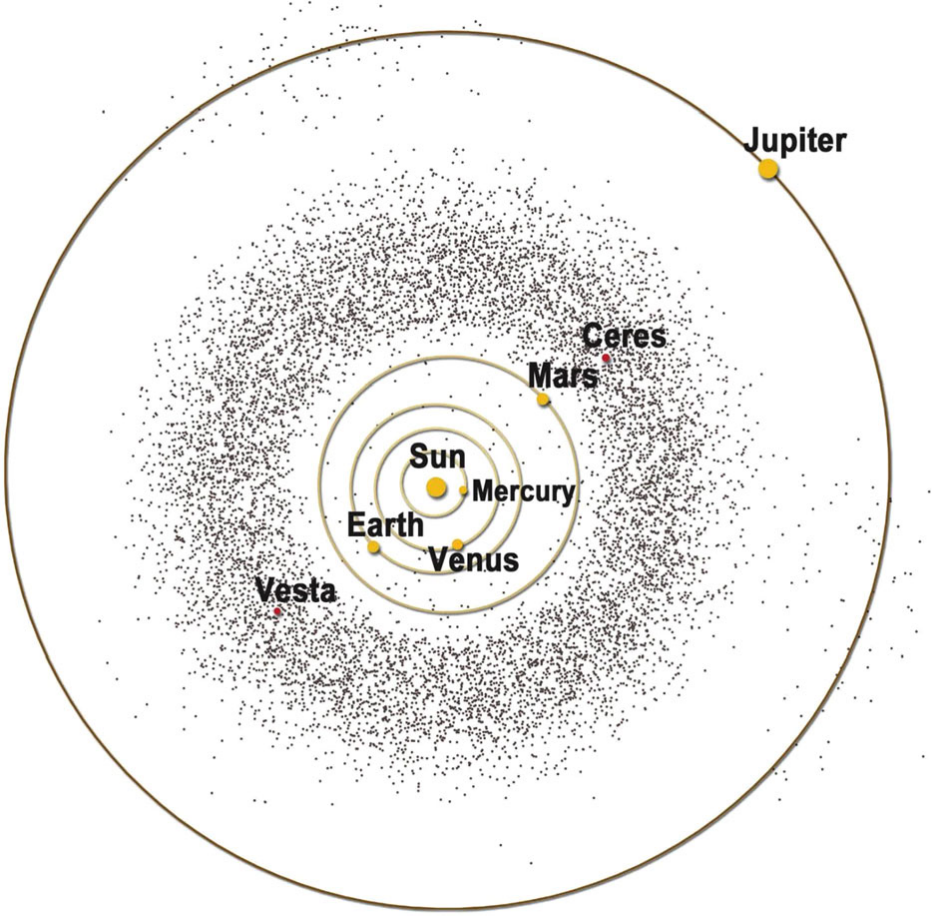
Artist’s depiction of the structure and location of the asteroid belt (2007). The locations of the Sun, Ceres, and Vesta are given. The orbits of Mercury, Venus, Earth, Mars, and Jupiter are also shown. Trojan asteroids, which are found at Jupiter’s distance from the Sun, are also displayed. Image credit: NASA/McREL

The asteroid belt is usually defined as the region lying roughly between the orbits of Mars and Jupiter (Fig. 1). One of the most significant features of the main belt is its low mass. It currently contains only ∼3% of the mass of the Moon. Estimates for the primordial mass of the asteroid belt range from between three and four orders of magnitude (Clement et al. 2019) to ∼3.5 times more massive (Levison et al. 2015) than its current mass. Significant mass loss from the belt likely took place at a very early stage in Solar System evolution, prior to Jupiter attaining its full size (Bottke et al. 2005; Clement et al. 2019).

At the broadest scale, the main belt has a distinct structure with the outer regions (∼2.8-3.3 AU) dominated by C-complex and P-type asteroids, whereas its inner regions (∼2.1-2.5 AU) contain a high percentage of S-complex asteroids (Gradie and Tedesco 1982; DeMeo and Carry 2014). However, on a more detailed scale, there is significant mixing of different spectral classes throughout the belt (DeMeo and Carry 2014).

**Fig. 2:**
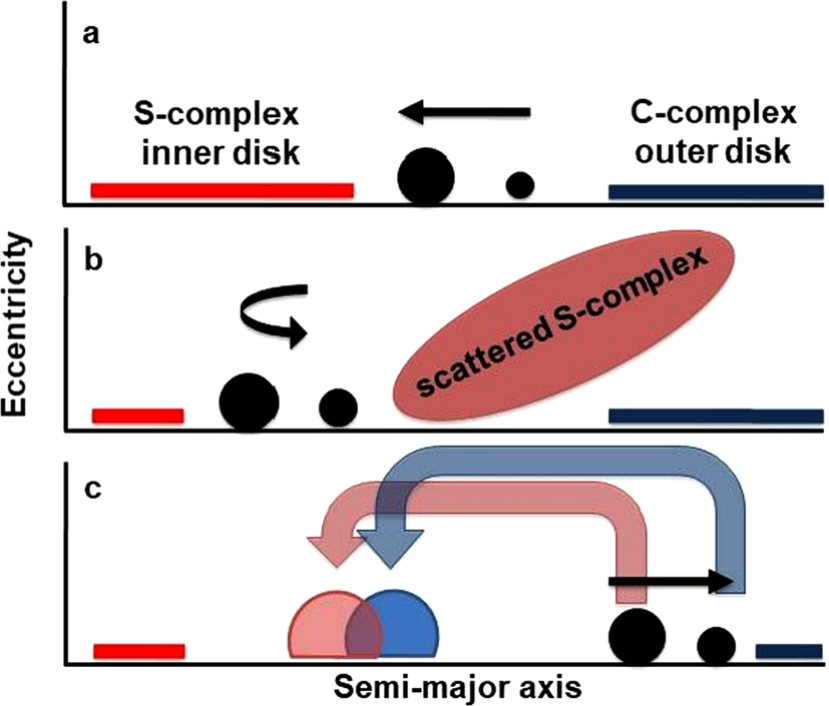
A cartoon of the “Grand Tack” model for the early evolution of the Solar System. The large dark circle is Jupiter and the smaller dark circle is Saturn. (**a**) Jupiter and Saturn are growing in a region outside the current location of the main belt with distinct populations of planetesimals in the inner and outer parts of the Solar System. (**b**) Jupiter and Saturn undergo gas-driven inward migration with many of the S-complex asteroids from the inner Solar System being scattered outwards. (**c**) Subsequent outwards migration of Jupiter and Saturn populates the inner main-belt region with S-complex asteroids that had originally been scattered outwards and then subsequently populates the outer margins of the main belt with C-complex asteroids, which are scattered inwards during the final stages of the gas giants’ migration. Plot used with the permission of Kevin Walsh

Dynamic models have been developed in part to explain the structure of the main belt and in part the small size of Mars. The “Grand Tack” model is based on the premise that prior to dispersion of the gas phase in the solar nebula, Jupiter and Saturn underwent an initial inwards migration with Jupiter reaching ∼1.5 AU, roughly the current location of Mars (Walsh et al. 2011, 2012) (Fig. 2) and then both bodies subsequently migrated outwards to their present positions. Prior to migration, Jupiter and Saturn were still in the final stages of accretion and a gas phase was present in the nebula. Migration would therefore have been early, only a few million years after the formation of the earliest nebular solids, calcium-aluminium-rich inclusions (CAIs). CAIs have mineralogies consistent with being early condensates from a hot gas of solar composition (Grossman 1972). The population of planetesimals at this early stage of Solar System evolution is considered to have been bimodal (Fig. 2a). Inwards of the gas giants (Jupiter and Saturn), planetesimals are believed to be essentially anhydrous S-complex bodies and outwards they were hydrated and volatile-rich (C-complex bodies). The initial inward migration of the gas giants to ∼1.5 AU scattered the S-complex planetesimals outwards, cleaning out the primordial belt region and truncating the disk (Fig. 2b). The restricted amount of material left at ∼1.5 AU is argued to be the reason for the relatively small size of Mars compared to the other terrestrial planets (Walsh et al. 2011). The inward migration of Jupiter and Saturn was then reversed when Saturn became captured in a 2:3 mean motion resonance (Pierens and Raymond 2011). As the outward migration took place, the gas giants would first have encountered the remnants of the S-complex bodies that had been scattered during the initial inward migration (Fig. 2c). These would then have been scattered back into the inner part of the main belt. As migration continued, the C-complex asteroids that would have populated the outer part of the Solar System would then have been encountered and a fraction of them scattered inwards to populate what became the outer portion of the current main belt (Walsh et al. 2011, 2012). The outward migration phase ended once the nebular gas had dissipated (Walsh et al. 2012).

The “Grand Tack” model provides a viable explanation for the origin and structure of the asteroid belt. It offers a coherent mechanism to explain how the main belt lost much of its primordial mass and also why it shows an overall trend from predominantly S-complex bodies at its inner margin to C-complex bodies at its outer margin. The “Grand Tack” model (Fig. 2) is inextricably linked to the concept of a bimodal Solar System in which materials that formed inwards of the “snow line” were distinctly different from those that formed outside of it (Lecar et al. 2006).

However, the degree of scattering of bodies in the “Grand Tack” model has not been tested with the analysis of samples derived from objects with known locations in the asteroid belt. Sample return from main-belt asteroids would allow mineralogical and isotopic compositions to be correlated with present distance from the Sun. Our meteorite collections contain evidence for between ∼100-150 parent bodies (e.g., Greenwood et al. 2020), but the “Grand Tack” model groups asteroids into only two types of general compositions (C- and S-complex bodies). Samples from main-belt bodies should allow the “Grand Tack” model to be refined to better duplicate the compositional and isotopic differences in the Solar System.

It is important to note that the “Grand Tack” is not the only recent model put forward to explain the present day structure of the main belt. The results of a dynamical simulation study undertaken by Raymond and Izidoro (2017) are consistent with the main belt forming empty and only acquiring mass as a consequence of scattering and drag processes from the inner terrestrial planet region and outer Solar System. Invoking a pebble accretion model, Kretke et al. (2017) reach similar conclusions.

Irrespective of whether the main belt evolved by significant mass loss or alternatively never had much mass is the first place, the concept of a compositionally bimodal Solar System is supported by significant geochemical evidence. We look in detail at the supporting evidence for a bimodal Solar System concept in the next section.

## Bimodality in the Early Solar System

**Fig. 3:**
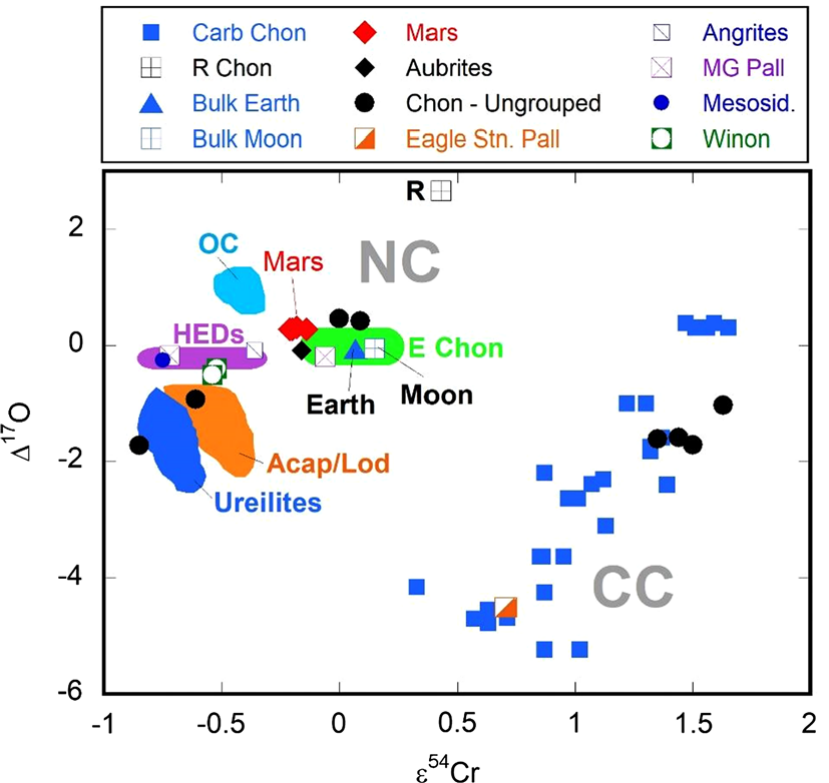
Plot of $\Delta ^{17}$O versus $\varepsilon ^{54}$Cr for a range of Solar System materials. This figure is modified from plots found in Warren (2011) and Greenwood et al. (2020). Two distinct clusters of data are present. The cluster in the upper left comprises the non-carbonaceous chondrite (NC) group and includes the ordinary chondrites (OC); enstatite chondrites (E Chon); R chondrites (R); Bulk Earth; Bulk Moon; Mars; howardites, eucrites, and diogenites (HEDs); main-group pallasites (MG Pall); aubrites; angrites; mesosiderites (Mesosid.); winonaites (Winon); and ureilites. The carbonaceous chondrite (CC) cluster in the lower right includes all the carbonaceous chondrites (Carb Chon), Eagle Station pallasites (Eagle Stn. Pall), and various ungrouped chondrites (Chon – Ungrouped). The NC and CC groups are separated by a well-defined gap, which is sometimes referred to as “The Warren Gap” (e.g., Voosen 2018) based on its initial identification by Warren (2011). By definition, the Earth falls at an $\varepsilon ^{54}$Cr value of 0 and a $\Delta ^{17}$O value of 0

Warren (2011), based on data from a range of earlier studies (e.g., Trinquier et al. 2007; Qin et al. 2010a), demonstrated that Solar System materials show a distinct bimodal distribution. with respect to a number of isotopic systems. This variation is clearly seen on a plot of $\Delta ^{17}$O versus $\varepsilon ^{54}$Cr (Fig. 3). Two distinct groupings are present. One cluster contains all the carbonaceous chondrites and a relatively minor subset of achondrites. The other consists of all other Solar System materials, including planetary-derived samples (Mars, Earth, Moon); ordinary, enstatite, and R chondrites; and a wide range of achondrites (main-group pallasites, howardites, eucrites, diogenites, ureilites, aubrites). Warren (2011) suggested that the carbonaceous chondrite (CC) group may represent material that accreted in the outer Solar System, whereas the non-carbonaceous chondrite (NC) group might be materials derived from the inner Solar System. The gap separating the NC and CC groups is sometimes referred to as “The Warren Gap” (e.g., Voosen 2018).

While the NC-CC dichotomy was originally identified with reference to the isotopes of a relatively limited number of elements (Cr, Ti, O, Ni, Mo, Mg) (Warren 2011; Budde et al. 2016; Van Kooten et al. 2016; Kruijer et al. 2017, 2020), this variation has now been documented for a much wider range of elements (Burkhardt et al. 2019). In addition to silicate-dominated types, iron meteorites also display a distinct bimodal distribution of nucleosynthetic anomalies (e.g., Budde et al. 2016).

With the notable exception of oxygen, isotopic mass-independent variation in extraterrestrial samples, not caused by spallation or radioactive decay, reflects nucleosynthetic processes in the feeder stars to the Solar System (Dauphas and Schauble 2016; Scott et al. 2018; Burkhardt et al. 2019; Nanne et al. 2019). Such anomalies are a reflection, at various scales, of the heterogeneous distribution of the presolar grains that carried these anomalies and which would have been derived from a range of stellar sources (Kruijer et al. 2020). The processes of mixing and homogenization that took place in the parental molecular cloud of the Solar System, and later within the solar protoplanetary disk, were not sufficient to erase these anomalies (Burkhardt et al. 2019; Nanne et al. 2019; Kruijer et al. 2020). In the case of oxygen, mass-independent variation may be the result of selective UV dissociation of CO, either in the presolar giant molecular cloud or the solar nebula (Clayton 2002; Yurimoto and Kuramoto 2004; Lyons and Young 2005). The oxygen isotope anomalies produced by this process may then have become locked into different phases, including water ice, gas, and dust. Preservation of these oxygen isotopic differences may also reflect incomplete homogenization in the protosolar nebula (Ireland et al. 2020).

Recent studies have invoked a change in the composition of the infalling material (Burkhardt et al. 2019; Nanne et al. 2019; Kruijer et al. 2020) to produce this isotopic dichotomy. CAIs are believed to have formed close to the proto-Sun and preserve the isotopic composition of the earliest Solar System solids. This material was enriched in nuclides produced in neutron-rich stellar environments and would have been transported outwards through viscous spreading. Later infalling material, assumed to be depleted in neutron-rich nuclides, would have tended to accumulate in the inner part of the disk and have diluted the isotopic signature within the NC region.

**Fig. 4:**
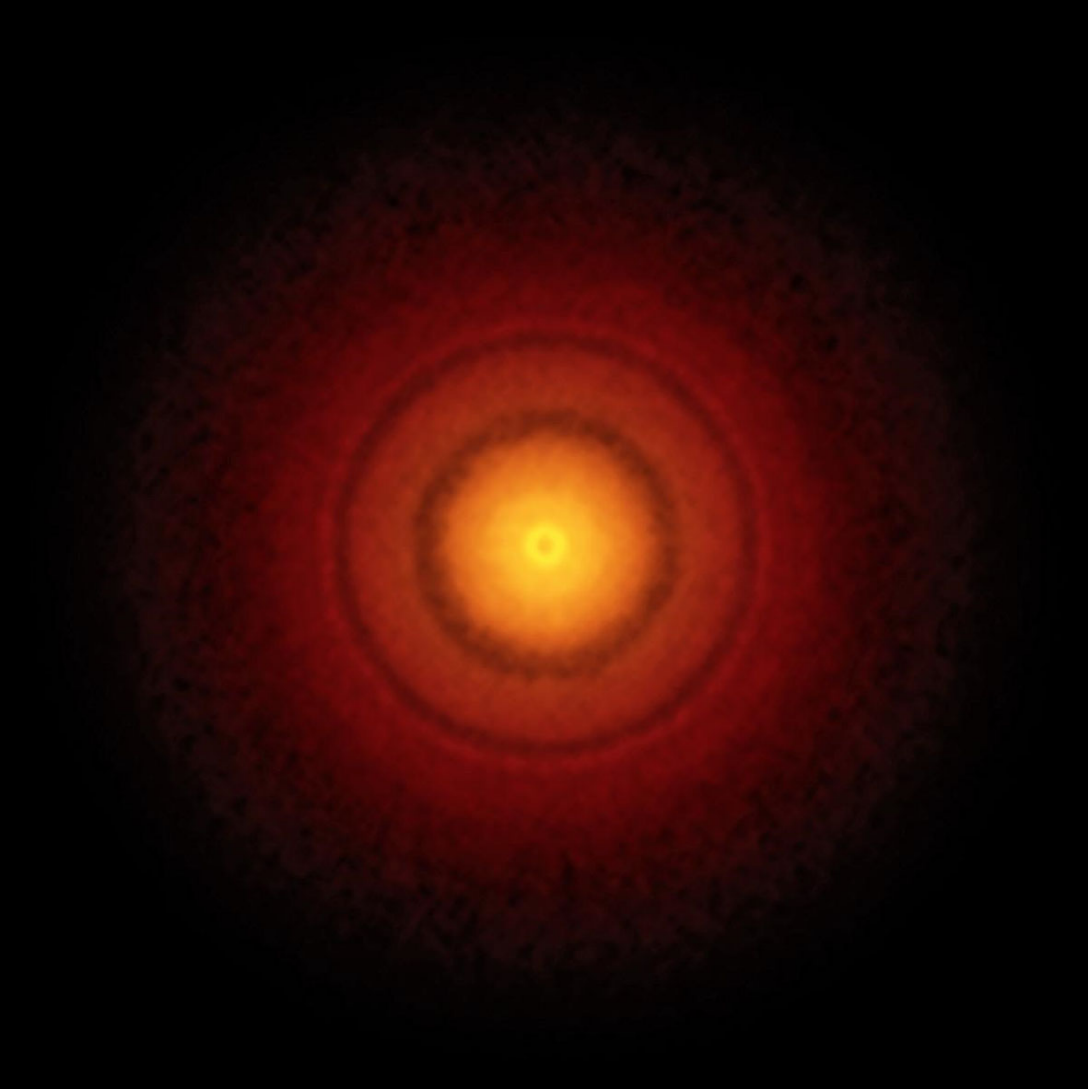
ALMA (Atacama Large Millimeter/submillimeter Array) image of protoplanetary disk around the T-Tauri star TW Hydrae (Andrews et al. 2016). This object is the closest T-Tauri star to the Solar System (196 light years away). The gaps in the disk are generally considered to be due to planets that are forming. Brasser and Mojzsis (2020) suggest that pressure maxima in the early Solar System protoplanetary disk may have resulted in a similar ring system. These pressure maxima may have prevented radial mixing. Image credit: S. Andrews (Harvard-Smithsonian CfA); B. Saxton (NRAO/AUI/NSF); ALMA (ESO/NAOJ/NRAO)

That Solar System materials should show significant variation with respect to a range of isotopic systems is not a novel finding and, particularly with respect to oxygen, has been well documented (Greenwood et al. 2017, 2020). However, the preservation of a distinct compositional gap between inner and outer Solar System-derived materials, as demonstrated by Warren (2011), is unexpected. The early Solar System was likely to have been a highly energetic environment, with considerable mixing taking place between different reservoirs of gas and dust (Misener et al. 2019). One explanation that has been advanced to explain the preservation of the NC-CC dichotomy is that it can be related to the early, rapid accretion of Jupiter, which then acted as a barrier between the inner and outer Solar System regions preventing complete isotopic homogenization within the disk (Kruijer et al. 2017, 2020). This scenario has been disputed by Brasser and Mojzsis (2020) who argue that Jupiter accreted at too slow a rate to have represented a significant barrier to mixing within the disk. Instead, Brasser and Mojzsis (2020) invoke a pressure maximum in the disk close to the present location of Jupiter and suggest that it was this feature, rather than the planet itself, which prevented significant mixing between the inner and outer Solar System regions. Brasser and Mojzsis (2020) speculate that the early Solar System may have had multiple rings, such as those observed by ALMA (Atacama Large Millimeter/submillimeter Array) in the disk around the young star TW Hydrae (Fig. 4) (Andrews et al. 2016). If this interpretation is correct then additional data clusters, over and above the dichotomy so far identified, might be present on isotopic plots such as shown in Fig. 3. The fact that additional clusters have not so far been identified may be a reflection of inadequate sampling of Solar System materials, an insufficient level of analytical resolution to identify such clusters, or alternatively these features do not actually exist.

Bimodality in Solar System materials is not just confined to isotopic anomalies. It has been well known for many decades that carbonaceous chondrites on the one hand and ordinary and enstatite chondrites on the other show distinctive and contrasting characteristics (Weisberg et al. 2006; Krot et al. 2014). Carbonaceous chondrites generally contain abundant CAIs, predominantly plot below the terrestrial fractionation line (TFL) on oxygen three-isotope plots and often show evidence of having accreted water ice into their parent bodies (e.g., Grimm and McSween 1989). In contrast, ordinary and enstatite chondrites generally have a relatively low CAI content, plot on or above the TFL, and show relatively anhydrous characteristics. Thus, a bimodal, inner versus outer Solar System dichotomy has always been evident in meteoritical studies, it is just that the new isotopic evidence has brought this concept into sharper focus (e.g., Scott et al. 2018).

The concept of bimodality in the meteorite record mirrors the overall structure of the Solar System itself, which is conventionally divided into the inner terrestrial planet region and the outer gas and ice giant region (Morbidelli et al. 2015). What lies between the two is the asteroid belt. Unsurprisingly, one of the most pertinent means of testing the bimodal Solar System concept would be to collect samples from both CC and NC main-belt asteroids. In theory, if enough samples were collected from different bodies with known locations, the asteroid belt could potentially be mapped both mineralogically and isotopically. However, there are clearly significant practical and economic difficulties in adopting such a global sampling approach. In Sects. 6 and 7, we look at various scenarios aimed at addressing the bimodal Solar System concept with sample return.

## Asteroid Families

The asteroid belt is dominated by groupings of asteroids in which individual members share similar proper orbital elements (Nesvorný et al. 2015). These clusterings are known as asteroidal families, with each family thought to be derived from the breakup of a single larger body or are derived from the debris ejected after a “large” impact on the parent body. With estimated ages that rarely exceed 1 billion years (Spoto et al. 2015), currently recognized families are significantly younger than the age of the Solar System and hence relatively ephemeral features of the asteroid belt, albeit very long-lived ones.

The advantage of an asteroid family member as a target for a sample return mission is that observations and samples collected at a single asteroid are relevant to a much larger number of related bodies. In particular, close-up exploration and subsequent collection of material from one member of a family would provide an independent verification of Earth-based observations (e.g., reflectance spectra) made on the family as a whole. There may be a huge scientific advantage in targeting a well-defined member of a well-constrained asteroid family (e.g., Nesvorný et al. 2015). For the purpose of testing the bimodal Solar System paradigm, amongst some of the most favourable targets could be C-complex and S-complex asteroid families.

## Meteorites and Their Relationship to Main-Belt Asteroids

Meteorites currently represent our principal source of extraterrestrial materials and so provide our best means of undertaking detailed mineralogical and isotopic studies of rocky planetary and asteroidal bodies. While our collections do contain materials derived from the Moon and Mars, most meteorites are asteroidal in origin. In terms of asteroidal sources, the vast majority of samples are likely derived from main-belt objects, rather than near-Earth asteroids. The cosmic-ray exposure ages of stony meteorites tend to be much longer than the dynamical lifetimes of bodies delivered to near-Earth space by resonances. These long cosmic-ray exposure ages for stony meteorites are consistent with entry into near-Earth space through slow Yarkovsky drift of meter-sized bodies derived from objects located anywhere in the main belt and not derivation directly from near-Earth bodies (e.g., Farinella et al. 1998; Bottke et al. 2002b). A cosmic-ray exposure age is the period of time that a surface (up to approximately one meter in depth) has been exposed to cosmic rays (e.g., Eugster et al. 2006) and is calculated by measuring the abundances of isotopes produced by cosmic-ray exposure. The Yarkovsky effect is the force acting on rotating objects due to the anisotropic emission of photons from blackbody radiation due to differences in temperature across the surface of the body.

There is a significant question mark about how representative the meteorites that arrive on Earth are of the material present in the main belt. In particular, atmospheric entry certainly acts as a filter preferentially destroying weak, friable objects at the expense of tough, strong meteoroids (Campbell-Brown 2019). That the meteorite flux is compositionally distinct from the micrometeorite flux is well established. Most micrometeorites have compositions that resemble CI and CM chondrites (Taylor et al. 2012) whereas these meteorite groups comprise only about 2% of meteorite falls (Burbine 2017). While this discrepancy is certainly not just a result of atmospheric entry processes, it serves to illustrate that major differences exist in the composition and, by implication, the likely origin of the different size fractions of extraterrestrial samples recovered on Earth.

Asteroids linked with “primitive” types of carbonaceous chondritic material predominate in the outer portion of the asteroid belt and so are less likely to arrive on Earth as recoverable samples. The Tagish Lake meteorite may be a notable exception due to its spectral similarity to D-type asteroids (Hiroi et al. 2001), which are abundant among Jupiter Trojans (e.g., Bendjoya et al. 2004). However, Granvik and Brown (2018) find that the orbit of Tagish Lake is most consistent with originating from the inner main-belt and not the outer main-belt. D-type asteroids are known to exist in the inner main-belt (DeMeo et al. 2014). Therefore, the parent body of Tagish Lake may have been scattered from its most likely original formation location in the outer belt to the inner main-belt.

There is an assumption that C-complex bodies primarily represent carbonaceous chondritic material and S-complex bodies primarily represent non-carbonaceous chondritic material such as ordinary chondrites. This assumption was validated for one body by the Hayabusa mission. The returned sample from S-complex NEA (25143) Itokawa has an LL-chondrite composition (Nakamura et al. 2011). However, the ureilite Almahata Sitta, which resides in the NC group (Qin et al. 2010b) of the Warren plot (Fig. 3), is derived from the C-complex NEA 2008 TC_3_ (Jenniskens et al. 2009), which was disrupted on impact with the atmosphere over the Sudan. This event illustrates the fact that it cannot be assumed that C-complex bodies will universally equate to the CC group. Targeting C-complex asteroids to recover material related to the CC group clearly needs to be done with care and with the benefit of significant input from remote sensing data (see Sect. 7 for further discussion).

Extraterrestrial samples that survive atmospheric entry will become altered to a variable extent due to interaction with the terrestrial environment. Meteorites that contain a significant metallic iron content are particularly susceptible to terrestrial weathering (Lee and Bland 2004; Greenwood et al. 2012). However, carbonaceous chondrites are also known to be affected by terrestrial alteration processes (e.g., Alexander et al. 2018). The well-studied CI chondrite Orgueil, which fell in France in 1864, has been noted to have been chemically altered (e.g., disappearance of ammonium sulfates) during its residence on Earth (Gounelle and Zolensky 2014). Direct collection and recovery of carbonaceous chondrite samples from the asteroid belt would help mitigate these alteration issues and is also likely to provide material that may not arrive on Earth as meteorites due to atmospheric entry processes.

## Asteroid Sample Return

### A Historical Perspective: Near-Earth Asteroid Sample Return (Hayabusa Mission)

The Hayabusa mission to Itokawa was the first mission to return samples from a near-Earth asteroid. Hayabusa was launched in May 2003 and rendezvoused with Itokawa in September 2005. In November 2005, Hayabusa landed on Itokawa to collect a sample. During the touchdown, a small pellet was supposed to be fired at the surface to cause the ejection of material into the sampling container; however, the pellet did not discharge. The hope was, as later confirmed, that during the encounter with the surface, small particles would have been ejected upward into the sampling container. In June 2010, the re-entry capsule returned to Earth, landing in the Australian desert. Approximately 700 grains from Itokawa have been catalogued (Okada et al. 2017). Hayabusa confirmed (Nakamura et al. 2011) the postulated compositional (Binzel et al. 2001) and isotopic similarity of Itokawa to LL chondrites.

Bulk and grain-specific mineralogy can be studied in extreme detail using a wide range of techniques, including scanning and analytical electron microscopy, electron probe analysis, electron backscattered diffraction, transmission electron microscopy, and Raman spectroscopy. Mass spectrometry can be used to measure both radiogenic and stable isotopic compositions. Such measurements provide information relevant to the formation region of the samples and the formation age of their parent body.

These studies returned a number of interesting results. Nakamura et al. (2011) determined that the fayalite and forsterite contents matched LL chondrites. Yurimoto et al. (2011) found that the Itokawa grains had an oxygen isotopic composition similar to LL chondrites. Noguchi et al. (2011) found that nanophase iron particles, the proposed cause of space weathering, were present in the grains. Jin and Bose (2019) measured the hydrogen isotopic composition and water content of a number of “anhydrous” minerals found in Itokawa grains and were able to derive the water content of the bulk silicate Itokawa parent body.

Studying returned particles has a number of advantages compared to studying whole meteorites. The particles would not have been exposed to the atmosphere during re-entry and would have a much more limited exposure to the terrestrial atmosphere. Individual grains from an asteroid’s surface can also be studied. Different grains may have distinct “personal histories” (a term coined by Tomoki Nakamura) on the asteroid due to experiencing different shock effects, space weathering, and/or thermal heating. The Hayabusa mission clearly demonstrates the benefit that sample return can have in providing extraterrestrial particles that would not otherwise have survived atmospheric entry without experiencing significant modification at best or, more likely, complete destruction.

### The Benefits of Combining Sample Return with Detailed Spacecraft Characterization Studies

Sample return from an asteroid would allow extraterrestrial material to be studied within a geologic context. When a meteorite is recovered on Earth, there is essentially no information available on where the sample originated, apart from some fireball trajectory information for a few relatively limited events (e.g., Spurný et al. 2012). However, when a sample is recovered directly from an asteroid, the location of the obtained material from the particular object is “known.” Prior to retrieval of the sample, the spacecraft undertakes detailed analysis and observations of the asteroid’s surface. These measurements provide critical geologic context and help to define the optimal sampling location. The terrain of the recovered sample and its relationship to different geologic features (e.g., craters, plains) on the surface can be established. The contextual information provided by the spacecraft’s remote sensing measurements, coupled with the subsequent results of detailed laboratory studies once material has been returned to Earth, constitutes a very powerful approach likely to yield important scientific results.

Sample return from main-belt asteroids is also important because it allows extraterrestrial material to be studied that may sample the surfaces of meteorite parent bodies and/or the oldest surviving planetesimals in the Solar System. Morbidelli et al. (2009) has argued that the minimum size of the initial planetesimals in our Solar System was ∼100 km, which would mean that ∼200 original planetesimals are possibly currently intact in the main belt.

The capabilities of Earth-based laboratory equipment still dwarf the capabilities of spacecraft instruments. Meteoritic material can often be analysed at scales of tens of nanometers (e.g., Kebukawa et al. 2019) to sub-nanometer (e.g., Parman et al. 2019), depending on the technique. Isotopic measurements of presolar grains of one micron or smaller can routinely be made in the laboratory (e.g., Davis 2011). Only bulk elemental compositions and estimated silicate mineralogies have currently been determined remotely for main-belt asteroids using spacecrafts (Burbine 2016). The use of well-calibrated standards allows a wide range of high-precision measurements to be obtained in the laboratory that are simply impossible using spacecraft-based techniques. So while sample-return missions may be expensive, the scientific yield from such missions more than offsets the cost.

### How to Return a Sample from a Main-Belt Asteroid

A number of studies have been done to investigate scenarios for main-belt sample return. Turtle et al. (1999) proposed the AMBASSADOR (A Main-Belt Asteroid Seismic study and Sample Acquisition to Determine meteorite ORigins) mission to study and return a sample from S-complex body (7) Iris (e.g., Gaffey et al. 1993; Noonan et al. 2019). The mission would consist of an orbiter and a lander. Material would be collected by two different methods. One would be a chipping device able to collect regolith using two circular blades that would drive material into a collection basket. Another would involve coring devices that are fired downward into the surface where recoil would return the cored material within the coring device back to the lander.

Sukhanov et al. (2001) proposed a low-cost sample-return mission to a main-belt asteroid. The spacecraft would eject a projectile that would strike the asteroid and then fly through the resulting dust cloud. Fragments would then be picked up by a collector on the spacecraft. This type of mission is a combination of the primary components of two cometary missions: Deep Impact and Stardust. Deep Impact sent an impactor to strike a comet Tempel 1 (9P/Tempel) and study the resulting crater and debris, while Stardust collected fragments of a comet’s (Wild 2) coma as it flew through it. Sukhanov et al. (2001) predicts that the collected mass would be ∼0.1-1 mg of material for their postulated sample-return mission to M-type (16) Psyche. Psyche is the current target of the Psyche mission (Elkins-Tanton et al. 2020), which will be launched in 2022. The orbiting spacecraft will determine the shape, the geology, the elemental composition, and the magnetic field of Psyche.

Deep Impact’s impactor was ∼360 kg in mass at impact. The impactor’s payload included a copper “cratering mass,” an Impactor Targeting Sensor (ITS), thrusters, a high-precision star tracker, and a radio receiver (Henderson and Blume 2015). Impactors proposed for main-belt asteroid sample-return missions are much smaller with masses of 10 kg or less (e.g., Morimoto et al. 2004). Stardust (Brownlee 2014) used a two-sided collector (∼0.1 m^2^) with containers filled with aerogel to slow down the fragments so they would not be vaporized on impact, plus aluminum foil where the fragments would actually vaporize. Besides fragments from the coma, Stardust also collected interstellar dust as it flew through the interstellar dust stream. However, collected grains may have experienced a short period of intense heating due to their high velocity when entering the aerogel (Roskosz et al. 2008). Approximately 1 mg of material was returned by Stardust. Stardust collected thousands of cometary dust particles plus a few chondrule fragments and CAIs (Westphal et al. 2017).

Morimoto et al. (2004) studied sample-return missions from main-belt asteroid families. Their proposed missions to the primarily S-complex Koronis family (Rivkin et al. 2011) and the multi-taxonomic Nysa-Polana complex (Walsh et al. 2013; Dykhuis and Greenberg 2015), respectively, also uses projectiles to impact each asteroid’s surface with the spacecraft flying through the resulting dust cloud. They propose that ∼10 kg projectiles would be released. Their target bodies have a wide variety of interpreted mineralogies.

Dachwald et al. (2008) studied whether a sample-return mission to C-complex (19) Fortuna using solar electric propulsion was feasible. The missions proposed by Dachwald et al. (2008) consist either of a lander with a sample-return vehicle powered by chemical propulsion or employ a spacecraft that samples the asteroid itself with a re-entry capsule to return the collected material to Earth by electric propulsion.

Sample-return missions to the C-complex dwarf planet (1) Ceres has also been the subject of a number of studies. Based on the discovery of active volatile-rich ejecta plumes on Ceres by the Herschel Space Observatory, Poncy et al. (2014) proposed a “low-cost” flyby mission to sample the plumes and return material to Earth. Fisher and Graham (2019) pointed out that Ceres represents a prime target for sample return and also advocated a “low-cost” option by utilizing a modified version of the OSIRIS-REx spacecraft configuration. GAUSS (Genesis of Asteroids and evolUtion of the Solar System) is a joint Chinese-European concept sample-return mission to Ceres, and the subject of a white paper (Shi et al. 2020) submitted to the European Space Agency’s Voyage 2050 initiative. Their study highlights the importance of returning samples from Ceres for our understanding of early Solar System processes and identifies various sampling sites and spacecraft configurations that might be used to accomplish this task. GAUSS is named after mathematician Johann Carl Friedrich Gauss (1777–1855) who was able to predict the position of Ceres after this body was “lost” (Cunningham 2016). Gauss’ technique to determine the preliminary orbit of a body using at least three observations is now called the Gauss method (Marsden 1985).

Not including the launch vehicle, the cost for the Stardust mission (launched in 1999) was approximately 200 million dollars, while the cost for OSIRIS-REx (launched in 2016) was approximately 800 million dollars. The cost of a main-belt sample-return mission is likely to be significantly more expensive than for OSIRIS-REx and so would probably exceed one billion dollars. However, as discussed in earlier sections, the scientific return from such a mission would be extremely high.

### What Objects Should We Sample?

A successful sample-return mission to any main-belt asteroid would represent a major engineering triumph with a huge scientific return. To investigate the isotopic bimodality of the Solar System, a number of bodies, expected to have different isotopic compositions, could be sampled. From our current knowledge, our best guess is that the C-complex bodies are the best analogues for CC material and that S-complex bodies are the best analogues for NC material. But what objects should be prioritized? Here we review potential target asteroids before setting out in Sect. 7 our preferred mission scenario for investigating the bimodal Solar System.

As discussed earlier, observations and samples collected from a member of an asteroid family will be relevant to a much larger number of related bodies. Two possible candidate C-complex families are the Nemesis (Carruba and Barletta 2019) and Adeona families (Carruba et al. 2003). Also of interest would be samples from members of a ∼4 billion year old family identified by Delbó et al. (2017) extending across the inner main-belt. Objects in this family tend to have very low albedos. Two possible S-complex family candidates are the Flora (Vernazza et al. 2008) and Koronis families (e.g., Rivkin et al. 2011).

**Fig. 5:**
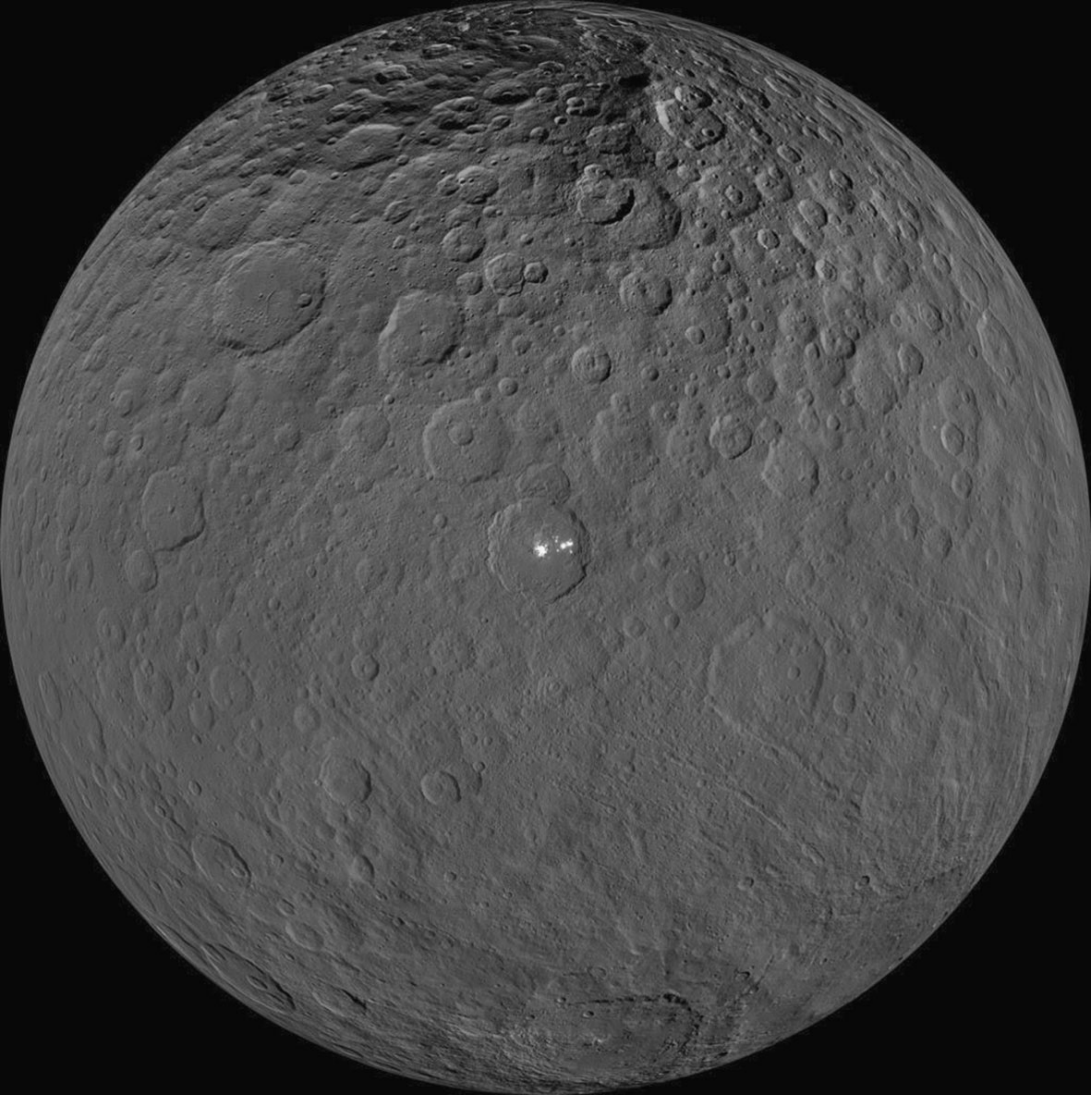
Orthographic projection of Ceres as seen by NASA’s Dawn spacecraft. The bright features in Occator crater are called faculae and appear to be due to hydrated magnesium sulfates (Nathues et al. 2015). Image credit: NASA/JPL-Caltech/UCLA/MPS/DLR/IDA

A number of relatively large well-studied bodies could also be possible targets. Dwarf planet Ceres ($a\approx 2.8$ AU), the second target of the Dawn mission (Fig. 5), has an interpreted mineralogy broadly consistent with carbonaceous chondrites (McSween et al. 2018) but no specific carbonaceous chondrite meteorite analogue has been identified. The interpreted presence of ammoniated hydrated silicates (King et al. 1992; Ammannito et al. 2016) may indicate Ceres’ formation in the outer Solar System where ammonia would have been stable. Isotopic analyses of material from Ceres would give considerable insight on possible formation locations. Another possible target could be (19) Fortuna ($a \approx 2.4$ AU). Fortuna has been proposed to be a possible parent body (Burbine 1998) for the CM2 chondrites due to spectral similarities in the visible and near-infrared and its location near the 3:1 mean-motion resonance.

Relatively large bodies that are expected to have non-carbonaceous isotopic compositions include (4) Vesta, (6) Hebe, and (16) Psyche. Vesta ($a \approx 2.4$ AU) has been studied in detail by the Dawn mission (Russell et al. 2013). Vesta has been linked with most HED (howardite, eucrite, diogenite) meteorites (McCord et al. 1970; Consolmagno and Drake 1977; McSween et al. 2013) due to spectral similarities. Vesta is the only large (∼525 km in diameter) body with an HED-like spectrum. However, a number of eucrites (e.g., NWA 011) have oxygen isotopic compositions that are anomalous compared to the values for most HEDs (Scott et al. 2009), implying multiple HED parent bodies. Sample return from Vesta could also “confirm” which HEDs are fragments of Vesta and whether it has the expected NC isotopic composition.

Asteroid (6) Hebe ($a \approx 2.4$ AU) would be another interesting target since it has been linked with both the H chondrites and IIE irons (Gaffey and Gilbert 1998) due to spectral similarities and Hebe’s location near the meteorite-supplying 3:1 resonance. However, Vernazza et al. (2014) has indicated from spectral studies that a number of bodies with interpreted mineralogies similar to H chondrites exist in the main belt. Sample return from Hebe would help us decipher whether Hebe is the parent body of the H chondrites and IIE irons.

Psyche ($a \approx 2.9$ AU) is commonly thought to be the core of a disrupted differentiated body (e.g., Ostro et al. 1985). Absorption features due to silicates have also been identified on the surface of Psyche (e.g., Hardersen et al. 2005). Fragments of Psyche could test whether Psyche is a core of a fragmented differentiated body and whether it has a NC or CC isotopic composition.

There are also a number of possible targets that have mineralogies that have perplexed ground-based researchers and may provide interesting insights on the history of the early Solar System. For example, asteroids such as (387) Aquitania ($a \approx 2.7$ AU) and (980) Anacostia ($a \approx 2.7$ AU) have long been known to have unusual spectral properties (Burbine et al. 1992). Their spectra appear consistent with a surface enriched in spinel-rich CAIs (Burbine et al. 1992; Sunshine et al. 2008). CAIs are the oldest dated material in the Solar System with the oldest CAI currently having an age of 4,568.22 ± 0.17 Ma (Bouvier and Wadhwa 2010). However, the implied CAI abundances for their surfaces are much higher than those found in chondrites. Sunshine et al. (2008) argues that these bodies are prime candidates for sample return since these objects might be some of the oldest bodies still existing today since they may have formed before the injection of radiogenic ^26^Al into the Solar System. The presence of significant concentrations of ^26^Al in these inclusions would be expected to have resulted in the melting of these bodies. Sample return from such bodies may allow CAIs that are older than currently known refractory inclusions to be studied. The implied high CAI abundances are consistent with a CC isotopic composition for these bodies.

## Preferred Mission Scenario: Return to Ceres

In the previous section, we looked at a wide range of potential target asteroids that could be sampled to investigate in further detail the bimodal Solar System concept. But as this is likely to be a relatively costly and technically-challenging mission, a coherent and well-defined strategy will be needed. Here we set out our preferred option for a sample-return mission to the main belt.

### Defining the Mission Strategy and Target Body

Based on the experience gained from previous sample-return missions, there are two distinct strategies that could be employed to successfully collect and return samples from the asteroid belt. These we term: (1) flyby and (2) direct sampling strategies. The flyby approach would involve visiting either a single, or preferably multiple asteroids with a spacecraft equipped with Deep Impact-style impactors. The impactors would be used to create a debris cloud that would be sampled by the spacecraft. By keeping the instrumentation to a minimum, costs could be reduced, thus enhancing the financial viability of such an approach. Visiting multiple target asteroids should provide a range of materials that hopefully would be representative of both the NC and CC groups.

However, there are also some downsides to the flyby approach. From past experience, the amount of material collected is likely to be limited and the complexity of visiting multiple targets increases the inherent risks of this type of mission. One particular problem, as exemplified by the Almahata Sitta meteorite (Sect. 5), is that not all C-complex asteroids are members of the CC group. Targeting smaller, less well-characterized C-complex main-belt asteroids leaves open the possibility that a flyby mission might fail to return CC group material.

While it would appear to be advantageous for a main-belt sample return mission investigating Solar System bimodality to return material from both NC- and CC-related bodies this concept may not stand up to more detailed scrutiny during the proposal review process. We already have a very large selection of NC-related materials and we actually live on the largest NC parent body. Rather than obtaining material from the asteroid belt to investigate inner Solar System evolution, we argue that a more fruitful approach and a better use of resources (time and money) would be to sample another large, inner Solar System body, such as Venus (Greenwood and Anand 2020). Venus would be expected to fall within the NC group.

In terms of the bimodal Solar System, we have a much poorer understanding of the CC group than the NC group. The amount of CC-related material delivered to Earth is highly biased and it is likely that friable and unconsolidated lithologies do not survive transit from the asteroid belt, for reasons already discussed. As a consequence, and paradoxically, rather than returning multiple samples from the main belt, the more targeted approach would be to return an unambiguous example of CC-related material. While returning material from the regions of Jupiter and beyond is unlikely to be feasible for the foreseeable future, one candidate body in the asteroid belt stands out as an exemplar of an outer Solar System icy-world. That object is Ceres, the largest body in the main belt. Ceres comprises one-third of the total mass of the main-belt region and is a possible refugee from the outer Solar System. Our preferred approach for investigating the bimodal Solar System would be direct sampling of a well-characterized location on Ceres. In the next section, we look in further detail at why sample return from Ceres would provide a first order scientific return capable of significantly improving our understanding of early Solar System evolution.

### Ceres After Dawn: An Icy World from the Outer Solar System

As a result of the Dawn mission, we now have a significant body of detailed topographic, geologic and compositional data for Ceres (Williams et al. 2018). A sample-return mission to Ceres would be visiting an already well characterized object, rather than a relatively unknown main-belt asteroid requiring detailed orbital observations prior to any attempt to collect material from its surface. The observations already completed by Dawn means that a sample-return mission to Ceres would need to carry a far less comprehensive suite of instruments than required for other main-belt bodies, thus ensuring significant cost savings.

The Dawn mission began orbiting Ceres in March 2015 (Russell et al. 2016) following its earlier phase of activity studying Vesta (Russell et al. 2013). Dawn continued to make orbital observations of Ceres until November 2018 when it ran out of fuel. Dawn’s observations resulted in a remarkable amount of information being learnt about the asteroid belt’s largest object. The surface was mapped at a resolution of 35 m/pixel during the LAMO (Low-Altitude Mapping Orbit) phase of operations (Williams et al. 2018). Compositional and mineralogical information was obtained with three instruments: the Framing Camera (FC), the Visible and InfraRed mapping spectrometer (VIR), and the Gamma Ray and Neutron Detector (GRaND) (Russell et al. 2006).

The results from the Dawn mission present a relatively coherent picture of Ceres as a carbonaceous chondrite-related, differentiated icy-world that has experienced a protracted history of water/rock interaction and alteration (McCord and Castillo-Rogez 2018). There is clear evidence on its surface in the form of bright deposits indicating geologically recent hydrothermal brine deposition (Scully et al. 2019). The possible presence of ammoniated phyllosilicates on its surface have been interpreted as indicating that Ceres may not have formed in its present location, but possibly further out in the trans-Neptunian disk (De Sanctis et al. 2015). This scenario is supported by the presence of ammonia ice on a number of trans-Neptunian objects, including (134340) Pluto, Pluto’s moon Charon, and (90482) Orcus (Brown and Calvin 2000; Barucci et al. 2008; DeMeo et al. 2015; Dalle Ore et al. 2019). De Sanctis et al. (2015) speculates that Ceres may have been implanted into the main belt during the “Grand Tack” migration (Walsh et al. 2011). On the basis of Dawn observations and our present understanding of the Solar System, there is currently very little doubt that Ceres is a bona fide representative of the CC group and as such would represent a target that is clearly relevant to the investigation of the bimodal Solar System concept.

Ceres bears some compositional resemblance to carbonaceous chondrites (CI/CM), but the match is not perfect (Castillo-Rogez et al. 2020). Ceres potentially contains more water and organics than CIs and CMs and the latter do not contain significant ammoniated phyllosilicates. The carbonate mineralogy detected on Ceres is significantly more diverse than found in CIs. In particular, sodium carbonate has never been found in a CI, but appears to be present on Ceres (Castillo-Rogez et al. 2020). The clear implication of these observations is that we do not have samples in our meteorite collections that are good matches to the mineralogy of Ceres. The high water content estimates for Ceres have been disputed by Zolotov (2020) who suggests instead that the body may have a high organic content of between 12 and 29 vol%. Based on the detection of ammoniated phyllosilicates and a potentially high organic content, it has been suggested that Ceres may be derived from further out in the Solar System than either the CIs or CMs (De Sanctis et al. 2015; Zolotov 2020).

### How and Where Would Samples Be Collected on Ceres?

**Fig. 6:**
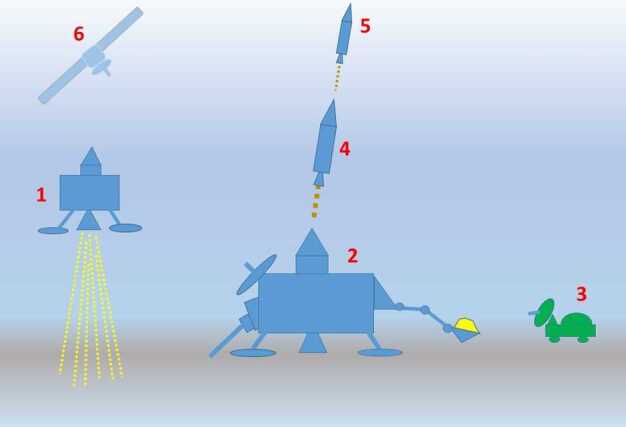
Schematic of a possible dedicated sample-return mission to Ceres. 1: Spacecraft arrives and is slowed during the landing phase by chemical propulsion. 2: Lander collects sample material. 3: Small reconnaissance rover has the ability to collect small amounts of additional material. 4 and 5: Two-stage rocket leaves base unit with samples once operations are complete. 6: For communication and imaging of the surface, a small orbiter would be required but would take no part in the return of samples to Earth

While it is beyond the scope of this study to identify a specific spacecraft configuration for returning a sample from Ceres, we do make some suggestions concerning a possible mission scenario. A flyby mission would not seem appropriate for the reasons discussed earlier. An orbiter/lander rendezvous maneuver following collection of material on the surface would also be a risky option. We would advocate a single lander approach to a well-scoped landing site (Fig. 6). Significant research has been done in developing strategies and equipment for returning both volatiles and non-volatiles from an ice-rich body back to Earth (e.g., Glavin et al. 2019). A small rover for reconnaissance studies could also be employed. Once the samples have been collected, a portion of the lander would then return to Earth without involving any transfer of material to another orbiting spacecraft. However, a small orbiter would be used for communication and imaging of the surface.

**Fig. 7:**
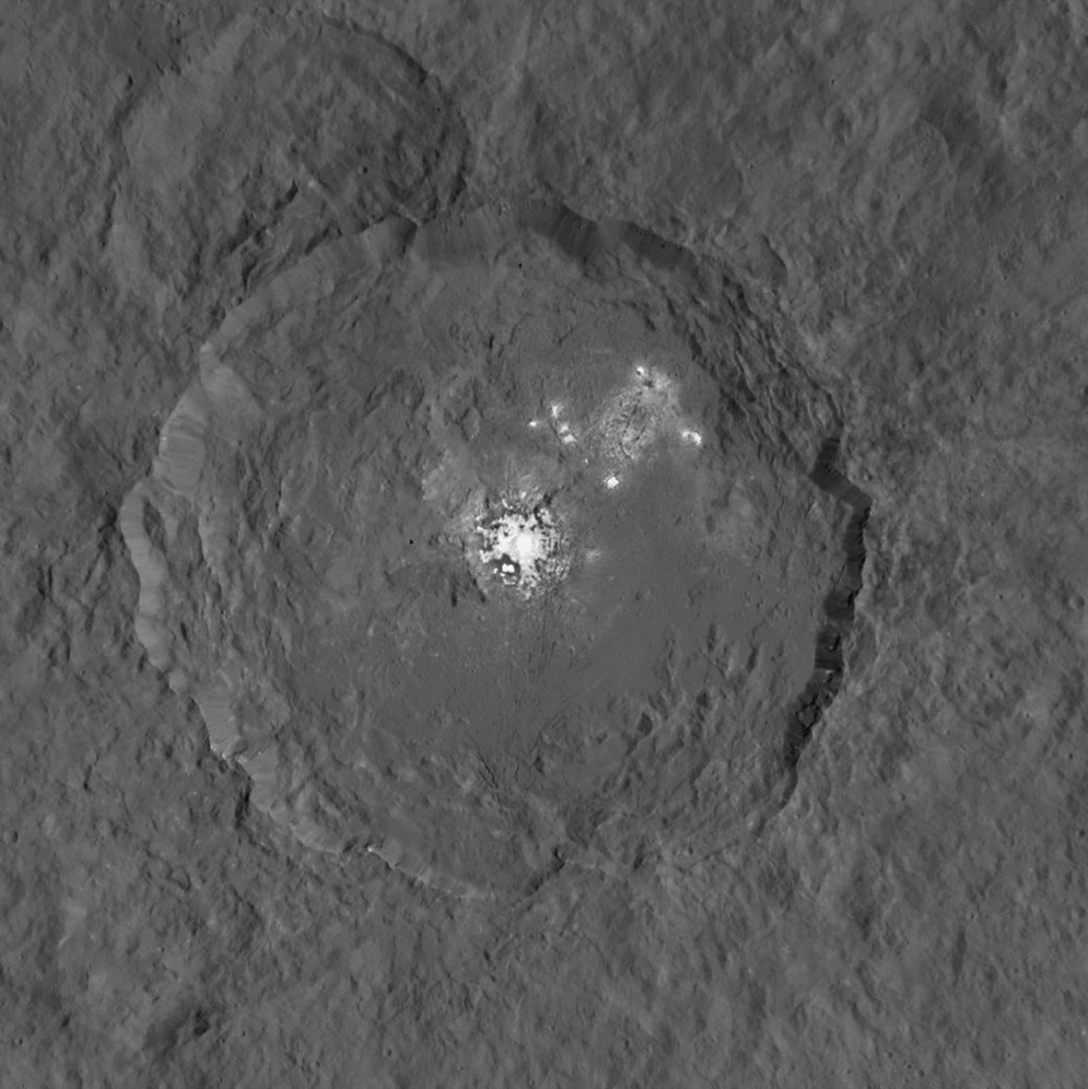
Close-up view of Occator crater on Ceres. The crater is approximately ∼90.5 km in diameter (Nathues et al. 2015) and is located at a latitude of 20° N and a longitude of 239° E. Image credit: NASA/JPL-Caltech/UCLA/MPS/DLR/IDA

In terms of specific locations on Ceres for the recovery of material, potential sampling sites would clearly need to be the subject of detailed evaluation. However, the Occator crater (Fig. 7), which has been the subject of a large number of detailed studies (e.g., Scully et al. 2019), does appear to be a particularly attractive possibility (Shi et al. 2020).

### Sample Analysis Strategy Back on Earth

A full suite of mineralogical and isotopic analyses would need to be undertaken on the returned material and consequently a Sample Analysis Plan (SAP) similar to that developed for the OSIRIS-REx mission (Lauretta et al. 2017) would need to be formulated and implemented. Material returned from Ceres would be expected to be hydrated and organic-rich (Raponi et al. 2018; Marchi et al. 2019; Zolotov 2020). Consequently, such samples would have major astrobiological implications (Castillo-Rogez et al. 2020). Although water ice is not expected to be close to the surface at equatorial latitudes, GRaND measurements suggests it is present in significant amounts at mid- to high-latitudes (Prettyman et al. 2017). Organic material appears to be particularly abundant on the surface of Ceres and likely to be of endogenous origin and not the result of meteorite infall, although this remains an issue of current debate (De Sanctis et al. 2019). Hydrogen isotopic analysis of organics and water (derived from hydrated silicates and possibly also ices) would provide additional constraints relevant to the origin of Ceres (Alexander et al. 2012).

In this study, we have focused on the bimodal variation in Solar System materials as exemplified by the range of isotopic systems (Fig. 3). However, there is an additional scenario raised by the study of Brasser and Mojzsis (2020), namely that the early Solar System was not just divided into two regions, but multiple ring-like regions were present (Fig. 4). It is possible that we now view the early Solar System as bimodal because we only receive samples from its two inner zones. If Ceres really did form much further out in the disk than anything we have so far in our meteorite collections, we could be in for an unexpected surprise. There is a real chance that Ceres is formed from material that we have never hitherto been able to sample. It is a tantalizing possibility.

## Conclusions

Sample return from the main belt is the logical next step in exploring the dichotomy present in Solar System materials. Isotopic studies indicate that a dichotomy existed in the early Solar System, with material from the inner region (NC group) being separated from outer region material (CC group) by a clear compositional gap on relevant variation diagrams. The interface between the NC and CC regions appears to be broadly coincident with the present location of the asteroid belt, which contains material derived from both groups. A significant range of compositionally diverse asteroids are available as targets of such a mission. A flyby mission using “Deep Impact”-style impactors to collect material from multiple targets has merits as a potential collection scenario. However, there are a number of downsides to this approach, not least of which is its potential complexity and consequent failure risks.

We argue that a sample-return mission to the asteroid belt does not necessarily have to return material from both the NC and CC groups to viably test the bimodal Solar System paradigm as the NC group is already abundantly available for study. Instead, there is overwhelming evidence that we have a very incomplete suite of CC-related samples. Based on our analysis, we advocate a dedicated sample-return mission to the dwarf planet Ceres as the best means of further exploring inherent Solar System variation. Ceres is an ice-rich world that may be a displaced trans-Neptunian object. We almost certainly do not have any samples in our current collections that would resemble material that could be brought back from Ceres. The rich heritage of data acquired by the Dawn mission makes a sample-return mission from Ceres logistically feasible at a realistic cost. No other potential target appears capable of providing as much insight into the early Solar System as Ceres. It should be given the highest priority by the international scientific community.

## References

[CR1] Alexander C.M.O’D., Bowden R., Fogel M.L., Howard K.T., Herd C.D.K., Nittler L.R. (2012). The provenances of asteroids, and their contributions to the volatile inventories of the terrestrial planets. Science.

[CR2] Alexander C.M.O’D., Greenwood R.C., Bowden R., Gibson J.M., Howard K.T., Franchi I.A. (2018). A multi-technique search for the most primitive CO chondrites. Geochim. Cosmochim. Acta.

[CR3] Ammannito E., DeSanctis M.C., Ciarniello M., Frigeri A., Carrozzo F.G., Combe J.-Ph., Ehlmann B.L., Marchi S., McSween H.Y., Raponi A., Toplis M.J., Tosi F., Castillo-Rogez J.C., Capaccioni F., Capria M.T., Fonte S., Giardino M., Jaumann R., Longobardo A., Joy S.P., Magni G., McCord T.B., McFadden L.A., Palomba E., Pieters C.M., Polanskey C.A., Rayman M.D., Raymond C.A., Schenk P.M., Zambon F., Russell C.T. (2016). Distribution of phyllosilicates on the surface of Ceres. Science.

[CR4] Andrews S.M., Wilner D.J., Zhu Z., Birnstiel T., Carpenter J.M., Pérez L.M., Bai X.-N., Öberg K.I., Hughes A.M., Isella A., Ricci L. (2016). Ringed substructure and a gap at 1 au in the nearest protoplanetary disk. Astrophys. J. Lett..

[CR5] Barucci M.A., Merlin F., Guilbert A., de Bergh C., Alvarez-Candal A., Hainaut O., Doressoundiram A., Dumas C., Owen T., Coradini A. (2008). Surface composition and temperature of the TNO Orcus. Astron. Astrophys..

[CR6] Bendjoya P., Cellino A., Di Martino M., Saba L. (2004). Spectroscopic observations of Jupiter Trojans. Icarus.

[CR7] Bierhaus E.B., Clark B.C., Harris J.W., Payne K.S., Dubisher R.D., Wurts D.W., Hund R.A., Kuhns R.M., Linn T.M., Wood J.L., May A.J., Dworkin J.P., Beshore E., Lauretta D.S. (2018). The OSIRIS-REx spacecraft and the Touch-and-Go Sample Acquisition Mechanism (TAGSAM). Space Sci. Rev..

[CR8] Binzel R.P., Rivkin A.S., Bus S.J., Sunshine J.M., Burbine T.H. (2001). MUSES-C target asteroid (25143) 1998 SF36: a reddened ordinary chondrite. Meteorit. Planet. Sci..

[CR9] Bottke W.F., Morbidelli A., Jedicke R., Petit J.-M., Levison H.F., Michel P., Metcalfe T.S. (2002). Debiased orbital and absolute magnitude distribution of the near-Earth objects. Icarus.

[CR10] Bottke W.F., Vokrouhlický D., Rubincam D.P., Brož M., Bottke W.F., Cellino A., Paolicchi P., Binzel R.P. (2002). The effect of Yarkovsky thermal forces on the dynamical evolution of asteroids and meteoroids. Asteroids III.

[CR11] Bottke W.F., Durda D.D., Nesvorný D., Jedicke R., Morbidelli A., Vokrouhlický D., Levison H. (2005). The fossilized size distribution of the main asteroid belt. Icarus.

[CR12] Bouvier A., Wadhwa M. (2010). The age of the Solar System redefined by the oldest Pb-Pb age of a meteoritic inclusion. Nat. Geosci..

[CR13] Bradley J.P., Holland H.D., Turekian K.K. (2014). Interplanetary dust particles. Meteorites and Cosmochemical Processes, Treatise on Geochemistry.

[CR14] Brasser R., Mojzsis S.J. (2020). The partitioning of the inner and outer Solar System by a structured protoplanetary disk. Nat. Astron..

[CR15] Brown M.E., Calvin W.M. (2000). Evidence for crystalline water and ammonia ices on Pluto’s satellite Charon. Science.

[CR16] Brown P.G., Hildebrand A.R., Zolensky M.E., Grady M., Clayton R.N., Mayeda T.K., Tagliaferri E., Spalding R., MacRae N.D., Hoffman E.L., Mittlefehldt D.W., Wacker J.F., Bird J.A., Campbell M.D., Carpenter R., Gingerich H., Glatiotis M., Greiner E., Mazur M.J., McCausland P.J.A., Plotkin H., Mazur T.R. (2000). The fall, recovery, orbit, and composition of the Tagish Lake meteorite: a new type of carbonaceous chondrite. Science.

[CR17] Brownlee D.E. (1985). Cosmic dust—collection and research. Annu. Rev. Earth Planet. Sci..

[CR18] Brownlee D. (2014). The Stardust mission: analyzing samples from the edge of the Solar System. Annu. Rev. Earth Planet. Sci..

[CR19] Budde G., Burkhardt C., Brennecka G.A., Fischer-Gödde M., Kruijer T.S., Kleine T. (2016). Molybdenum isotopic evidence for the origin of chondrules and a distinct genetic heritage of carbonaceous and non-carbonaceous meteorites. Earth Planet. Sci. Lett..

[CR20] Burbine T.H. (1998). Could G-class asteroids be the parent bodies of the CM chondrites?. Meteorit. Planet. Sci..

[CR21] Burbine T.H. (2016). Advances in determining asteroid chemistries and mineralogies. Chem. Erde.

[CR22] Burbine T.H. (2017). Asteroids: Astronomical and Geological Bodies.

[CR23] Burbine T.H., Gaffey M.J., Bell J.F. (1992). S-asteroids 387 Aquitania and 980 Anacostia: possible fragments of the breakup of a spinel-bearing parent body with CO3/CV3 affinities. Meteoritics.

[CR24] Burkhardt C., Dauphas N., Hans U., Bourdon B., Kleine T. (2019). Elemental and isotopic variability in Solar System materials by mixing and processing of primordial disk reservoirs. Geochim. Cosmochim. Acta.

[CR25] Campbell-Brown M.D. (2019). Meteoroid structure and fragmentation. Planet. Space Sci..

[CR26] Carruba V., Barletta W. (2019). The influence of Ceres on the dynamical evolution of the Zdenekhorsky/Nemesis asteroid family. Planet. Space Sci..

[CR27] Carruba V., Burns J.A., Bottke W., Nesvorný D. (2003). Orbital evolution of the Gefion and Adeona asteroid families: close encounters with massive asteroids and the Yarkovsky effect. Icarus.

[CR28] Castillo-Rogez J.C., Neveu M., Scully J.E.C., House C.H., Quick L.C., Bouquet A., Miller K., Bland M., De Sanctis M.C., Ermakov A., Hendrix A.R., Prettyman T.H., Raymond C.A., Russell C.T., Sherwood B.E., Young E. (2020). Astrobiological target and possible ocean world. Astrobiology.

[CR29] Clayton R.N. (2002). Solar System: self-shielding in the solar nebula. Nature.

[CR30] Clement M.S., Raymond S.N., Kaib N.A. (2019). Excitation and depletion of the asteroid belt in the early instability scenario. Astron. J..

[CR31] Consolmagno G.J., Drake M.J. (1977). Composition and evolution of the eucrite parent body: evidence from rare Earth elements. Geochim. Cosmochim. Acta.

[CR32] Cunningham C.J. (2016). Discovery of the First Asteroid, Ceres.

[CR33] Dachwald B., Seboldt W., Loeb H.W., Schartner K.H. (2008). Main-belt asteroid sample return mission using solar electric propulsion. Acta Astronaut..

[CR34] Dalle Ore C.M., Cruikshank D.P., Protopapa S., Scipioni F., McKinnon W.B., Cook J.C., Grundy W.M., Schmitt B., Stern S.A., Moore J.M., Verbiscer A., Parker A.H., Singer K.N., Umurhan O.M., Weaver H.A., Olkin C.B., Young L.A., Ennico K., New Horizons Surface Composition Science Theme Team (2019). Detection of ammonia on Pluto’s surface in a region of geologically recent tectonism. Sci. Adv..

[CR35] Dauphas N., Schauble E.A. (2016). Mass fractionation laws, mass-independent effects, and isotopic anomalies. Annu. Rev. Earth Planet. Sci..

[CR36] Davis A.M. (2011). Stardust in meteorites. Proc. Natl. Acad. Sci..

[CR37] De Sanctis M.C., Ammannito E., Raponi A., Marchi S., McCord T.B., McSween H.Y., Capaccioni F., Capria M.T., Carrozzo F.G., Ciarniello M., Longobardo A., Tosi F., Fonte S., Formisano M., Frigeri A., Giardino M., Magni G., Palomba E., Turrini D., Zambon F., Combe J.-P., Feldman W., Jaumann R., McFadden L.A., Pieters C.M., Prettyman T., Toplis M., Raymond C.A., Russell C.T. (2015). Ammoniated phyllosilicates with a likely outer Solar System origin on (1) Ceres. Nature.

[CR38] De Sanctis M.C., Vinogradoff V., Raponi A., Ammannito E., Ciarniello M., Carrozzo F.G., De Angelis S., Raymond C.A., Russell C.T. (2019). Characteristics of organic matter on Ceres from VIR/Dawn high spatial resolution spectra. Mon. Not. R. Astron. Soc..

[CR39] Delbó M., Walsh K., Bolin B., Avdellidou C., Morbidelli A. (2017). Identification of a primordial asteroid family constrains the original planetesimal population. Science.

[CR40] DeMeo F.E., Carry B. (2014). Solar System evolution from compositional mapping of the asteroid belt. Nature.

[CR41] DeMeo F.E., Binzel R.P., Carry B., Polishook D., Moskovitz N.A. (2014). Unexpected D-type interlopers in the inner main belt. Icarus.

[CR42] DeMeo F.E., Dumas C., Cook J.C., Carry B., Merlin F., Verbiscer A.J., Binzel R.P. (2015). Spectral variability of Charon’s 2.21-μm feature. Icarus.

[CR43] Devillepoix H.A.R., Sansom E.K., Bland P.A., Towner M.C., Cupák M., Howie R.M., Jansen-Sturgeon T., Cox M.A., Hartig B.A.D., Benedix G.K., Paxman J.P. (2018). The Dingle Dell meteorite: a Halloween treat from the main belt. Meteorit. Planet. Sci..

[CR44] Dykhuis M.J., Greenberg R. (2015). Collisional family structure within the Nysa-Polana complex. Icarus.

[CR45] Elkins-Tanton L.T., Asphaug E., Bell III J.F., Bercovici H., Bills B., Binzel R., Bottke W.F., Dibb S., Lawrence D.J., Marchi S., McCoy T.J., Oran R., Park R.S., Peplowski P.N., Polanskey C.A., Prettyman T.H., Russell C.T., Schaefer L., Weiss B.P., Wieczorek M.A., Williams D.A., Zuber M.T. (2020). Observations, meteorites, and models: a preflight assessment of the composition and formation of (16) Psyche. J. Geophys. Res.: Planets.

[CR46] Eugster O., Herzog G.F., Marti K., Caffee M.W., Lauretta D.S., McSween H.Y. (2006). Irradiation records, cosmic-ray exposure ages, and transfer times of meteorites. Meteorites and the Early Solar System II.

[CR47] Farinella P., Vokrouhlický D., Hartmann W.K. (1998). Meteorite delivery via Yarkovsky orbital drift. Icarus.

[CR48] Fisher K.R., Graham L.D. (2019). Ceres: a prime target for robotic sample return and future human exploration. 50th Lunar and Planetary Science Conference.

[CR49] Gaffey M.J., Gilbert S.L. (1998). Asteroid 6 Hebe: the probable parent body of the H-type ordinary chondrites and the IIE iron meteorites. Meteorit. Planet. Sci..

[CR50] Gaffey M.J., Bell J.F., Brown R.H., Burbine T.H., Piatek J., Reed K.L., Chaky D.A. (1993). Mineralogic variations within the S-type asteroid class. Icarus.

[CR51] Gladman B., Migliorini F., Morbidelli A., Zappalà V., Michel P., Cellino A., Froeschlé Ch., Levison H., Bailey M., Duncan M. (1997). Dynamical lifetimes of objects injected into asteroid belt resonances. Science.

[CR52] Glavin D.P., Squyres S.W., Chu P.C., Gerakines P.A., Yamada K., Parker J.E., Wegel D.C., Peabody H.L., Houghton M.B., Aslam S., Gorius N., Quilligan G., Thorpe R.S., Spring J., Zacny K.A., Gorevan S., Okazaki S., Satoh Y., Maru Y., Nakao T., Kukita A., Shimoda T., Yamawaki T., Hayes A.G., Lauretta D.S., Messenger S., Nakamura-Messenger K., Mumma M.J., Milam S.N., Lunine J.I., Mitchell J.L., Hasegawa M.M., Takahara P.M., Nakamura T., Dworkin J.P., Clemett S., Blake G.A., Soderblom J.M., Furukawa Y., Kimura Y., Takigawa A., CAESAR Project Team (2019). The CAESAR New Frontiers mission: 3. Sample acquisition and preservation. 50th Lunar and Planetary Science Conference.

[CR53] Gounelle M., Zolensky M.E. (2014). The Orgueil meteorite: 150 years of history. Meteorit. Planet. Sci..

[CR54] Gradie J., Tedesco E. (1982). Compositional structure of the asteroid belt. Science.

[CR55] Granvik M., Brown P. (2018). Identification of meteorite source regions in the Solar System. Icarus.

[CR56] Greenwood R.C., Anand M. (2020). What is the oxygen isotope composition of Venus? The scientific case for sample return from Earth’s “sister” planet. Space Sci. Rev..

[CR57] Greenwood R.C., Franchi I.A., Gibson J.M., Benedix G.K. (2012). Oxygen isotope variation in primitive achondrites: the influence of primordial, asteroidal and terrestrial processes. Geochim. Cosmochim. Acta.

[CR58] Greenwood R.C., Burbine T.H., Miller M.F., Franchi I.A. (2017). Melting and differentiation of early-formed asteroids: the perspective from high precision oxygen isotope studies. Chem. Erde.

[CR59] Greenwood R.C., Burbine T.H., Franchi I.A. (2020). Linking asteroids and meteorites to the primordial planetesimal population. Geochim. Cosmochim. Acta.

[CR60] Grimberg A., Baur H., Bochsler P., Bühler F., Burnett D.S., Hays C.C., Heber V.S., Jurewicz A.J.G., Wieler R. (2006). Solar wind neon from Genesis: implications for the lunar noble gas record. Science.

[CR61] Grimm R.E., McSween H.Y. (1989). Water and the thermal evolution of carbonaceous chondrite parent bodies. Icarus.

[CR62] Grossman L. (1972). Condensation in the primitive solar nebula. Geochim. Cosmochim. Acta.

[CR63] Hardersen P.S., Gaffey M.J., Abell P.A. (2005). Near-IR spectral evidence for the presence of iron-poor orthopyroxenes on the surfaces of six M-type asteroids. Icarus.

[CR64] Hartmann W.K., Davis D.R. (1975). Satellite-sized planetesimals and lunar origin. Icarus.

[CR65] Henderson M., Blume W. (2015). Deep Impact—a review of the world’s pioneering hypervelocity impact mission. Procedia Eng..

[CR66] Hiroi T., Zolensky M.E., Pieters C.M. (2001). The Tagish Lake meteorite: a possible sample from a D-type asteroid. Science.

[CR67] Ireland T.R., Avila J., Greenwood R.C., Hicks L.J., Bridges J.C. (2020). Oxygen isotopes and sampling of the Solar System. Space Sci. Rev..

[CR68] Jenniskens P., Shaddad M.H., Numan D., Elsir S., Kudoda A.M., Zolensky M.E., Le L., Robinson G.A., Friedrich J.M., Rumble D., Steele A., Chesley S.R., Fitzsimmons A., Duddy S., Hsieh H.H., Ramsay G., Brown P.G., Edwards W.N., Tagliaferri E., Boslough M.B., Spalding R.E., Dantowitz R., Kozubal M., Pravec P., Borovicka J., Charvat Z., Vaubaillon J., Kuiper J., Albers J., Bishop J.L., Mancinelli R.L., Sandford S.A., Milam S.N., Nuevo M., Worden S.P. (2009). The impact and recovery of asteroid 2008 TC_3_. Nature.

[CR69] Jin Z., Bose M. (2019). New clues to ancient water on Itokawa. Sci. Adv..

[CR70] Kebukawa Y., Kobayashi H., Urayama N., Baden N., Kondo M., Zolensky M.E., Kobayashi K. (2019). Nanoscale infrared imaging analysis of carbonaceous chondrites to understand organic-mineral interactions during aqueous alteration. Proc. Natl. Acad. Sci..

[CR71] King T.V.V., Clark R.N., Calvin W.M., Sherman D.M., Brown R.H. (1992). Evidence for ammonium-bearing minerals on Ceres. Science.

[CR72] Kleine T., Budde G., Burkhardt C., Kruijer T.S., Worsham E.A., Morbidelli A., Nimmo F. (2020). The non-carbonaceous—carbonaceous meteorite dichotomy. Space Sci. Rev..

[CR73] Kretke K.A., Bottke W.F., Levison H.F., Kring D.A. (2017). Mixing of the asteroid belt due to the formation of the giant planets. Accretion: Building New Worlds Conference.

[CR74] Krot A.N., Keil K., Scott E.R.D., Goodrich C.A., Weisberg M.K., Holland H.D., Turekian K.K. (2014). Classification of meteorites and their genetic relationships. Meteorites and Cosmochemical Processes, Treatise on Geochemistry.

[CR75] Kruijer T.S., Burkhardt C., Budde G., Kleine T. (2017). Age of Jupiter inferred from the distinct genetics and formation times of meteorites. Proc. Natl. Acad. Sci..

[CR76] Kruijer T.S., Kleine T., Borg L.E. (2020). The great isotopic dichotomy of the early Solar System. Nat. Astron..

[CR77] Lauretta D.S., Balram-Knutson S.S., Beshore E., Boynton W.V., Drouet d’Aubigny C., DellaGiustina D.N., Enos H.L., Golish D.R., Hergenrother C.W., Howell E.S., Bennett C.A., Morton E.T., Nolan M.C., Rizk B., Roper H.L., Bartels A.E., Bos B.J., Dworkin J.P., Highsmith D.E., Lorenz D.A., Lim L.F., Mink R., Moreau M.C., Nuth J.A., Reuter D.C., Simon A.A., Bierhaus E.B., Bryan B.H., Ballouz R., Barnouin O.S., Binzel R.P., Bottke W.F., Hamilton V.E., Walsh K.J., Chesley S.R., Christensen P.R., Clark B.E., Connolly H.C., Crombie M.K., Daly M.G., Emery J.P., McCoy T.J., McMahon J.W., Scheeres D.J., Messenger S., Nakamura-Messenger K., Righter K., Sandford S.A. (2017). OSIRIS-REx: sample return from asteroid (101955) Bennu. Space Sci. Rev..

[CR78] Lauretta D.S., DellaGiustina D.N., Bennett C.A., Golish D.R., Becker K.J., Balram-Knutson S.S., Barnouin O.S., Becker T.L., Bottke W.F., Boynton W.V., Campins H., Clark B.E., Connolly H.C., Drouet d’Aubigny C.Y., Dworkin J.P., Emery J.P., Enos H.L., Hamilton V.E., Hergenrother C.W., Howell E.S., Izawa M.R.M., Kaplan H.H., Nolan M.C., Rizk B., Roper H.L., Scheeres D.J., Smith P.H., Walsh K.J., Wolner C.W.V., The OSIRIS-REx Team (2019). The unexpected surface of asteroid (101955) Bennu. Nature.

[CR79] Lecar M., Podolak M., Sasselov D., Chiang E. (2006). On the location of the snow line in a protoplanetary disk. Astrophys. J..

[CR80] Lee M.R., Bland P.A. (2004). Mechanisms of weathering of meteorites recovered from hot and cold deserts and the formation of phyllosilicates. Geochim. Cosmochim. Acta.

[CR81] Levison H.F., Kretke K.A., Walsh K.J., Bottke W.F. (2015). Growing the terrestrial planets from the gradual accumulation of submeter-sized objects. Proc. Natl. Acad. Sci..

[CR82] Lunar Sample Preliminary Examination Team (1969). Preliminary examination of lunar samples from Apollo 11. Science.

[CR83] Lyons J.R., Young E.D. (2005). CO self-shielding as the origin of oxygen isotope anomalies in the early solar nebula. Nature.

[CR84] Marchi S., Raponi A., Prettyman T.H., De Sanctis M.C., Castillo-Rogez J., Raymond C.A., Ammannito E., Bowling T., Ciarniello M., Kaplan H., Palomba E., Russell C.T., Vinogradoff V., Yamashita N. (2019). An aqueously altered carbon-rich Ceres. Nat. Astron..

[CR85] Marsden B.G. (1985). Initial orbit determination: the pragmatist’s point of view. Astron. J..

[CR86] McCord T.B., Castillo-Rogez J.C. (2018). Ceres’s internal evolution: the view after Dawn. Meteorit. Planet. Sci..

[CR87] McCord T.B., Adams J.B., Johnson T.V. (1970). Asteroid Vesta: spectral reflectivity and compositional implications. Science.

[CR88] McKeegan K.D., Kallio A.P.A., Heber V.S., Jarzebinski G., Mao P.H., Coath C.D., Kunihiro T., Wiens R.C., Nordholt J.E., Moses R.W., Reisenfeld D.B., Jurewicz A.J.G., Burnett D.S. (2011). The oxygen isotopic composition of the Sun inferred from captured solar wind. Science.

[CR89] McSween H.Y., Binzel R.P., de Sanctis M.C., Ammannito E., Prettyman T.H., Beck A.W., Reddy V., Le Corre L., Gaffey M.J., McCord T.B., Raymond C.A., Russell C.T., The Dawn Science Team (2013). Dawn; the Vesta-HED connection; and the geologic context for eucrites, diogenites, and howardites. Meteorit. Planet. Sci..

[CR90] McSween H.Y., Emery J.P., Rivkin A.S., Toplis M.J., Castillo-Rogez J.C., Prettyman T.H., De Sanctis M.C., Pieters C.M., Raymond C.A., Russell C.T. (2018). Carbonaceous chondrites as analogs for the composition and alteration of Ceres. Meteorit. Planet. Sci..

[CR91] Meteoritical Bulletin Database (2020). https://www.lpi.usra.edu/meteor/

[CR92] Misener W., Krijt S., Ciesla F.J. (2019). Tracking dust grains during transport and growth in protoplanetary disks. Astrophys. J..

[CR93] Morbidelli A., Bottke W.F., Nesvorný D., Levison H.F. (2009). Asteroids were born big. Icarus.

[CR94] Morbidelli A., Lambrechts M., Jacobson S., Bitsch B. (2015). The great dichotomy of the Solar System: small terrestrial embryos and massive giant planet cores. Icarus.

[CR95] Morimoto M., Yamakawa H., Yoshikawa M., Abe M., Yano H. (2004). Trajectory design of multiple asteroid sample return missions. Adv. Space Res..

[CR96] Nakamura T., Noguchi T., Tanaka M., Zolensky M.E., Kimura M., Tsuchiyama A., Nakato A., Ogami T., Ishida H., Uesugi M., Yada T., Shirai K., Fujimura A., Okazaki R., Sandford S.A., Ishibashi Y., Abe M., Okada T., Ueno M., Mukai T., Yoshikawa M., Kawaguchi J. (2011). Itokawa dust particles: a direct link between S-type asteroids and ordinary chondrites. Science.

[CR97] Nanne J.A.M., Nimmo F., Cuzzi J.N., Kleine T. (2019). Origin of the non-carbonaceous–carbonaceous meteorite dichotomy. Earth Planet. Sci. Lett..

[CR98] Nathues A., Hoffmann M., Schaefer M., Le Corre L., Reddy V., Platz T., Cloutis E.A., Christensen U., Kneiss T., Li J.-Y., Mengel K., Schmedemann N., Schaefer T., Russell C.T., Applin D.M., Buczkowski D.L., Izawa M.R.M., Keller H.U., O’Brien D.P., Pieters C.M., Raymond C.A., Ripken J., Schenk P.M., Schmidt B.E., Sierks H., Sykes M.V., Thangjam G.S., Vincent J.-B. (2015). Sublimation in bright spots on (1) Ceres. Nature.

[CR99] National Research Council (2011). Vision and Voyages for Planetary Science in the Decade 2013–2022.

[CR100] Nesvorný D., Brož M., Carruba V., Michel P., DeMeo F.E., Bottke W.F. (2015). Identification and dynamical properties of asteroid families. Asteroids IV.

[CR101] Noguchi T., Nakamura T., Kimura M., Zolensky M.E., Tanaka M., Hashimoto T., Konno M., Nakato A., Ogami T., Fujimura A., Abe M., Yada T., Mukai T., Ueno M., Okada T., Shirai K., Ishibashi Y., Okazaki R. (2011). Incipient space weathering observed on the surface of Itokawa dust particles. Science.

[CR102] Noguchi T., Ohashi N., Tsujimoto S., Mitsunari T., Bradley J.P., Nakamura T., Toh S., Stephan T., Iwata N., Imae N. (2015). Cometary dust in Antarctic ice and snow: past and present chondritic porous micrometeorites preserved on the Earth’s surface. Earth Planet. Sci. Lett..

[CR103] Noonan J.W., Reddy V., Harris W.M., Bottke W.F., Sanchez J.A., Furfaro R., Brown Z., Fernandes R., Kareta T., Lejoly C., Nallapu R.T., Khan Niazi H., Slick L.R., Schatz L., Sharkey B.N.L., Springmann A., Angle G., Bailey L., Acuna D.D., Lewin C., Marchese K., Meshel M., Quintero N., Tatum K., Wilburn G. (2019). Search for the H chondrite parent body among the three largest S-type asteroids: (3) Juno, (7) Iris, and (25) Phocaea. Astron. J..

[CR104] Okada T., Binzel R.P., Connolly H.C., Yada T., Ohtsuki K. (2017). Special issue “Science of Solar System materials examined from Hayabusa and future missions (II)”. Earth Planets Space.

[CR105] Ostro S.J., Campbell D.B., Shapiro I.I. (1985). Mainbelt asteroids: dual-polarization radar observations. Science.

[CR106] Parman S., Jacobsen S., Petaev M., Akey A. (2019). Atom probe tomography of opaque assemblage in Allende CAI. 50th Lunar and Planetary Science Conference.

[CR107] Pierens A., Raymond S.N. (2011). Two phase, inward-then outward migration of Jupiter and Saturn in the gaseous solar nebula. Astron. Astrophys..

[CR108] Poncy J., Fontdecaba J., Couzin P. (2014). A robust mission concept for a low-cost Ceres plume sample return. EPSC Abstracts Vol. 9.

[CR109] Prettyman T.H., Yamashita N., Toplis M.J., McSween H.Y., Schörghofer N., Marchi S., Feldman W.C., Castillo-Rogez J., Forni O., Lawrence D.J., Ammannito E., Ehlmann B.L., Sizemore H.G., Joy S.P., Polanskey C.A., Rayman M.D., Raymond C.A., Russell C.T. (2017). Extensive water ice within Ceres’ aqueously altered regolith: evidence from nuclear spectroscopy. Science.

[CR110] Qin L., Alexander C.M.O’D., Carlson R.W., Horan M.F., Yokoyama T. (2010). Contributions to chromium isotope variations of meteorites. Geochim. Cosmochim. Acta.

[CR111] Qin L., Rumble D., Alexander C.M.O’D., Carlson R.W., Jenniskens P., Shaddad M.H. (2010). The chromium isotopic composition of Almahata Sitta. Meteorit. Planet. Sci..

[CR112] Raponi A., De Sanctis M.C., Frigeri A., Ammannito E., Ciarniello M., Formisano M., Combe J.-P., Magni G., Tosi F., Carrozzo F.G., Fonte S., Giardino M., Joy S.P., Polanskey C.A., Rayman M.D., Capaccioni F., Capria M.T., Longobardo A., Palomba E., Zambon F., Raymond C.A., Russell C.T. (2018). Variations in the amount of water ice on Ceres’ surface suggest a seasonal water cycle. Sci. Adv..

[CR113] Raymond S.N., Izidoro A.Z. (2017). The empty primordial asteroid belt. Sci. Adv..

[CR114] Rivkin A.S., Thomas C.A., Trilling D.E., Enga M.-t., Grier J.A. (2011). Ordinary chondrite-like colors in small Koronis family members. Icarus.

[CR115] Roskosz M., Leroux H., Watson H.C. (2008). Thermal history, partial preservation and sampling bias recorded by Stardust cometary grains during their capture. Earth Planet. Sci. Lett..

[CR116] Russell C.T., Capaccioni F., Coradini A., Christensen U., De Sanctis M.C., Feldman W.C., Jaumann R., Keller H.U., Konopliv A., McCord T.B., McFadden L.A., McSween H.Y., Mottola S., Neukum G., Pieters C.M., Prettyman T.H., Raymond C.A., Smith D.E., Sykes M.V., Williams B., Zuber M.T. (2006). Dawn discovery mission to Vesta and Ceres: present status. Adv. Space Res..

[CR117] Russell C.T., Raymond C.A., Jaumann R., McSween H.Y., De Sanctis M.C., Nathues A., Prettyman T.H., Ammannito E., Reddy V., Preusker F., O’Brien D.P., Marchi S., Denevi B.W., Buczkowski D.L., Pieters C.M., McCord T.B., Li J.-Y., Mittlefehldt D.W., Combe J.-P., Williams D.A., Hiesinger H., Yingst R.A., Polanskey C.A., Joy S.P. (2013). Dawn completes its mission at 4 Vesta. Meteorit. Planet. Sci..

[CR118] Russell C.T., Raymond C.A., Ammannito E., Buczkowski D.L., De Sanctis M.C., Hiesinger H., Jaumann R., Konopliv A.S., McSween H.Y., Nathues A., Park R.S., Pieters C.M., Prettyman T.H., McCord T.B., McFadden L.A., Mottola S., Zuber M.T., Joy S.P., Polanskey C., Rayman M.D., Castillo-Rogez J.C., Chi P.J., Combe J.P., Ermakov A., Fu R.R., Hoffmann M., Jia Y.D., King S.D., Lawrence D.J., Li J.-Y., Marchi S., Preusker F., Roatsch T., Ruesch O., Schenk P., Villarrea M.N., Yamashita N. (2016). Dawn arrives at Ceres: exploration of a small, volatile-rich world. Science.

[CR119] Saal A.E., Hauri E.H., Cascio M.L., Van Orman J.A., Rutherford M.C., Cooper R.F. (2008). Volatile content of lunar volcanic glasses and the presence of water in the Moon’s interior. Nature.

[CR120] Scott E.R.D., Greenwood R.C., Franchi I.A., Sanders I.S. (2009). Oxygen isotopic constraints on the origin and parent bodies of eucrites, diogenites, and howardites. Geochim. Cosmochim. Acta.

[CR121] Scott E.R.D., Krot A.N., Sanders I.S. (2018). Isotopic dischotomy among meteorites and its bearing on the protoplanetary disk. Astrophys. J..

[CR122] Scully J.E.C., Bowling T., Bu C., Buczkowski D.L., Longobardo A., Nathues A., Neesemann A., Palomba E., Quick L.C., Raponi A., Ruesch O., Schenk P.M., Stein N.T., Thomas E.C., Russell C.T., Castillo-Rogez J.C., Raymond C.A., Jaumann R., Dawn Science Team (2019). The formation and evolution of Ceres’ Occator crater. Icarus.

[CR123] Sears D.W.G. (1998). The case for rarity of chondrules and calcium-aluminum-rich inclusions in the early Solar System and some implications for astrophysical models. Astrophys. J..

[CR124] Shaddad M.H., Jenniskens P., Numan D., Kudoda A.M., Elsir S., Riyad I.F., Ali A.E., Alameen M., Alameen N.M., Eid O., Osman A.T., Abubaker M.I., Yousif M., Chesley S.R., Chodas P.W., Albers J., Edwards W.N., Brown P.G., Kuiper J., Friedrich J.M. (2010). The recovery of asteroid 2008 TC_3_. Meteorit. Planet. Sci..

[CR125] Shi X., Castillo-Rogez J., Hsieh H., Hui H., Ip W.-H., Lei H., Li J.-Y., Tosi F., Zhou L., Agarwal J., Barucci A., Beck P., Campo Bagatin A., Capaccioni F., Coates A., Cremonese G., Duffard R., Jaumann R., Jones G., Grande M., Kallio E., Lin Y., Mousis O., Nathues A., Oberst J., Showman A., Sierks H., Ulamec S., Wang M. (2020). GAUSS – A Sample Return Mission to Ceres ESA Voyage 2050 White Paper.

[CR126] Spoto F., Milani A., Knežević Z. (2015). Asteroid family ages. Icarus.

[CR127] Spurný P., Bland P.A., Shrbený L., Borovička J., Ceplecha Z., Singelton A., Bevan A.W.R., Vaughan D., Towner M.C., McClafferty T.P., Toumi R., Deacon G. (2012). The Bunburra Rockhole meteorite fall in SW Australia: Fireball trajectory, luminosity, dynamics, orbit, and impact position from photographic and photoelectric records. Meteorit. Planet. Sci..

[CR128] Sukhanov A.A., Durão O., Lazzaro D. (2001). Low-cost main-belt asteroid sample return. J. Spacecr. Rockets.

[CR129] Sunshine J.M., Connolly H.C., McCoy T.J., Bus S.J., La Croix L.M. (2008). Ancient asteroids enriched in refractory inclusions. Science.

[CR130] Taylor S., Matrajt G., Guan Y. (2012). Fine-grained precursors dominate the micrometeorite flux. Meteorit. Planet. Sci..

[CR131] Trinquier A., Birck J.-L., Allègre C.J. (2007). Widespread ^54^Cr heterogeneity in the inner Solar System. Astrophys. J..

[CR132] Turtle E.P., Minitti M.E., Cohen B.A., Chabot N.L., Tourbier D., Bachman C., Brock J., Foerstner R., Hoppa G.V., Kay J., Lewicki C.A., Mastrapa R.M.E., Patel J., Sherman N., Spitale J.N., Rivkin A.S., Trilling D.E., Villegas D., Weitz C.M., The JPL Advanced Projects Design Team (1999). AMBASSADOR: asteroid sample return mission to 7 Iris. Acta Astronaut..

[CR133] Usui T., Bajo K.-i., Fujiya W., Furukawa Y., Koike M., Miura Y.N., Sugahara H., Tachibana S., Takano Y., Kuramoto K. (2020). The importance of Phobos sample return for understanding the Mars-Moon system. Space Sci. Rev..

[CR134] Van Kooten E.M.M.E., Wielandt D., Schiller M., Nagashima K., Thomen A., Larsen K.K., Olsen M.B., Nordlund Å., Krot A.N., Bizzarro M. (2016). Isotopic evidence for primordial molecular cloud material in metal-rich carbonaceous chondrites. Proc. Natl. Acad. Sci..

[CR135] Vernazza P., Binzel R.P., Thomas C.A., DeMeo F.E., Bus S.J., Rivkin A.S., Tokunaga A.T. (2008). Compositional differences between meteorites and near-Earth asteroids. Nature.

[CR136] Vernazza P., Zanda B., Binzel R.P., Hiroi T., DeMeo F.E., Birlan M., Hewins R., Ricci L., Barge P., Lockhart M. (2014). Multiple and fast: the accretion of ordinary chondrite parent bodies. Astrophys. J..

[CR137] Voosen P. (2018). Meteorite divide points to solar system chaos. Science.

[CR138] Wada K., Grott M., Michel P., Walsh K.J., Barucci A.M., Biele J., Blum J., Ernst C.M., Grundmann J.T., Gundlach B., Hagermann A., Hamm M., Jutzi M., Kim M.-J., Kührt E., Le Corre L., Libourel G., Lichtenheldt R., Maturilli A., Messenger S.R., Michikami T., Miyamoto H., Mottola S., Müller T., Nakamura A.M., Nittler L.R., Ogawa K., Okada T., Palomba E., Sakatani N., Schröder S.E., Senshu H., Takir D., Zolensky M.E., International Regolith Science Group (IRSG) in Hayabusa2 project (2018). Asteroid Ryugu before the Hayabusa2 encounter. Prog. Earth Planet. Sci..

[CR139] Walsh K.J., Morbidelli A., Raymond S.N., O’Brien D.P., Mandell A.M. (2011). A low mass for Mars from Jupiter’s early gas-driven migration. Nature.

[CR140] Walsh K.J., Morbidelli A., Raymond S.N., O’Brien D.P., Mandell A.M. (2012). Populating the asteroid belt from two parent source regions due to the migration of giant planets—“The Grand Tack”. Meteorit. Planet. Sci..

[CR141] Walsh K.J., Delbó M., Bottke W.F., Vokrouhlický D., Lauretta D.S. (2013). Introducing the Eulalia and new Polana asteroid families: re-assessing primitive asteroid families in the inner main-belt. Icarus.

[CR142] Warren P.H. (2011). Stable-isotopic anomalies and the accretionary assemblage of the Earth and Mars: a subordinate role for carbonaceous chondrites. Earth Planet. Sci. Lett..

[CR143] Watanabe S., Hirabayashi M., Hirata N., Hirata Na., Noguchi R., Shimaki Y., Ikeda H., Tatsumi E., Yoshikawa M., Kikuchi S., Yabuta H., Nakamura T., Tachibana S., Ishihara Y., Morota T., Kitazato K., Sakatani N., Matsumoto K., Wada K., Senshu H., Honda C., Michikami T., Takeuchi H., Kouyama T., Honda R., Kameda S., Fuse T., Miyamoto H., Komatsu G., Sugita S., Okada T., Namiki N., Arakawa M., Ishiguro M., Abe M., Gaskell R., Palmer E., Barnouin O.S., Michel P., French A.S., McMahon J.W., Scheeres D.J., Abell P.A., Yamamoto Y., Tanaka S., Shirai K., Matsuoka M., Yamada M., Yokota Y., Suzuki H., Yoshioka K., Cho Y., Tanaka S., Nishikawa N., Sugiyama T., Kikuchi H., Hemmi R., Yamaguchi T., Ogawa N., Ono G., Mimasu Y., Yoshikawa K., Takahashi T., Takei Y., Fujii A., Hirose C., Iwata T., Hayakawa M., Hosoda S., Mori O., Sawada H., Shimada T., Soldini S., Yano H., Tsukizaki R., Ozaki M., Iijima Y., Ogawa K., Fujimoto M., Ho T.-M., Moussi A., Jaumann R., Bibring J.-P., Krause C., Terui F., Saiki T., Nakazawa S., Tsuda Y. (2019). Hayabusa2 arrives at the carbonaceous asteroid 162173 Ryugu—a spinning top–shaped rubble pile. Science.

[CR144] Weisberg M.K., McCoy T.J., Krot A.N., Lauretta D.S., McSween H.Y. (2006). Systematics and evaluation of meteorite classification. Meteorites and the Early Solar System II.

[CR145] Westphal A.J., Bridges J.C., Brownlee D.E., Butterworth A.L., De Gregorio B.T., Dominguez G., Flynn G.J., Gainsforth Z., Ishii H.A., Joswiak D., Nittler L.R., Ogliore R.C., Palma R., Pepin R.O., Stephan T., Zolensky M.E. (2017). The future of Stardust science. Meteorit. Planet. Sci..

[CR146] Williams D.A., Buczkowski D.L., Mest S.C., Scully J.E.C., Platz T., Kneissl T. (2018). Introduction: the geologic mapping of Ceres. Icarus.

[CR147] Wood J.A., Dickey J.S., Marvin U.B., Powell B.N. (1970). Lunar anorthosites. Science.

[CR148] Yurimoto H., Kuramoto K. (2004). Molecular cloud origin for the oxygen isotope heterogeneity in the Solar System. Science.

[CR149] Yurimoto H., Abe K.-i., Abe M., Ebihara M., Fujimura A., Hashiguchi M., Hashizume K., Ireland T.R., Itoh S., Katayama J., Kato C., Kawaguchi J., Kawasaki N., Kitajima F., Kobayashi S., Meike T., Mukai T., Nagao K., Nakamura T., Naraoka H., Noguchi T., Okazaki R., Park C., Sakamoto N., Seto Y., Takei M., Tsuchiyama A., Uesugi M., Wakaki S., Yada T., Yamamoto K., Yoshikawa M., Zolensky M.E. (2011). Oxygen isotopic compositions of asteroidal materials returned from Itokawa by the Hayabusa mission. Science.

[CR150] Zolensky M.E., Zega T.J., Yano H., Wirick S., Westphal A.J., Weisberg M.K., Weber I., Warren J.L., Velbel M.A., Tsuchiyama A., Tsou P., Toppani A., Tomioka N., Tomeoka K., Teslich N., Taheri M., Susini J., Stroud R., Stephan T., Stadermann F.J., Snead C.J., Simon S.B., Simionovici A., See T.H., Robert F., Rietmeijer F.J.M., Rao W., Perronnet M.C., Papanastassiou D.A., Okudaira K., Ohsumi K., Ohnishi I., Nakamura-Messenger K., Nakamura T., Mostefaoui S., Mikouchi T., Meibom A., Matrajt G., Marcus M.A., Leroux H., Lemelle L., Le L., Lanzirotti A., Langenhorst F., Krot A.N., Keller L.P., Kearsley A.T., Joswiak D., Jacob D., Ishii H., Harvey R., Hagiya K., Grossman L., Grossman J.N., Graham G.A., Gounelle M., Gillet P., Genge M.J., Flynn G., Ferroir T., Fallon S., Fakra S., Ebel D.S., Rong Dai Z., Cordier P., Clark B., Chi M., Butterworth A.L., Brownlee D.E., Bridges J.C., Brennan S., Brearley A., Bradley J.P., Bleuet P., Bland P.A., Bastien R. (2006). Mineralogy and petrology of comet 81P/Wild 2 nucleus samples. Science.

[CR151] Zolotov M.Yu. (2020). The composition and structure of Ceres’ interior. Icarus.

